# Antipathogenic properties and applications of low-dimensional materials

**DOI:** 10.1038/s41467-021-23278-7

**Published:** 2021-06-23

**Authors:** Z. L. Shaw, Sruthi Kuriakose, Samuel Cheeseman, Michael D. Dickey, Jan Genzer, Andrew J. Christofferson, Russell J. Crawford, Chris F. McConville, James Chapman, Vi Khanh Truong, Aaron Elbourne, Sumeet Walia

**Affiliations:** 1 School of Engineering, RMIT University, Melbourne, Australia; 2 Functional Materials and Microsystems Research Group, MicroNano Research Facility, RMIT University, Melbourne, Australia; 3 School of Science, RMIT University, Melbourne, VIC Australia; 4 Department of Chemical and Biomolecular Engineering, North Carolina State University, Raleigh, NC USA; 5 Institute for Frontier Materials, Deakin University, Geelong, Victoria, 3220 Australia

**Keywords:** Antimicrobials, Biomedical materials, Two-dimensional materials

## Abstract

A major health concern of the 21^st^ century is the rise of multi-drug resistant pathogenic microbial species. Recent technological advancements have led to considerable opportunities for low-dimensional materials (LDMs) as potential next-generation antimicrobials. LDMs have demonstrated antimicrobial behaviour towards a variety of pathogenic bacterial and fungal cells, due to their unique physicochemical properties. This review provides a critical assessment of current LDMs that have exhibited antimicrobial behaviour and their mechanism of action. Future design considerations and constraints in deploying LDMs for antimicrobial applications are discussed. It is envisioned that this review will guide future design parameters for LDM-based antimicrobial applications.

## Abstract (web summary)

Antimicrobial resistance is a growing global problem and low dimensional materials have emerged as a potential solution. Here, the authors review the progress which has been made on low dimensional antimicrobials looking at the materials synthesis, modes of action and currently applications.

## Introduction

Antimicrobial agents play a vital role in the prevention of pathogenic bacterial and fungal infections, accounting for a predicted 95% reduction in mortality rates since their introduction in the late 1940s^1–4^. However, in recent times, over-prescription and misuse of antibiotics in medical, veterinary and agricultural industries, combined with the rapid evolution of microbial species, has contributed to the emergence of antimicrobial resistance (AMR) in pathogenic microbes^1,2^. Globally, single and multi-drug resistant (MDR) microbes are responsible for ~700,000 deaths each year^1^. The World Health Organisation (WHO), has named several concerning drug resistant, pathogenic bacterial strains prevalent in global healthcare settings, including MDR tuberculosis (MDR-TB), carbapenem-resistant *Enterobacteriaceae* (CRE), methicillin-resistant S*taphylococcus aureus* (MRSA) and MDR *Neisseria gonorrhoeae*^1,3,4^. Resistant fungal strains, including MDR *Candida auris*, are also becoming increasingly resistant to all available antifungal treatments^2^. Without the development of new antimicrobial agents, the WHO estimates that by 2050, approximately 10 million people will die annually from previously treatable infections^5^. The predicted economic impact of AMR suggests it could be worse than the 2008 global financial crisis^4^, costing over US$100 trillion in healthcare^5^.

In response, considerable research has been conducted in an attempt to combat these infections^6–9^. Unfortunately, the development of new antimicrobial drugs has faltered in recent years, with only two new classes of antibiotics receiving approval from international regulatory agencies in the past 20 years^4^ with neither being effective against Gram-negative bacteria. This has driven research towards the development of alternative antimicrobial nanomaterials including polymers^6^, particles^7^ and surface coatings^8^. One of the first nanomaterials that demonstrated high antipathogenic abilities is silver nanoparticles and has subsequently received an extensive amount of attention in the research community^10,11^. More recently, low-dimensional materials (LDMs) have gained significant interest as potential antimicrobial agents^12,13^. These materials are categorised as zero-dimensional (0D)^14^, one-dimensional (1D)^15^ or two-dimensional (2D)^16^ nanomaterials based on their respective dimensionality. These materials have demonstrated favourable properties that have led to their use in electronics and sensing^17,18^. A more comprehensive understanding of the underlying properties of LDMs, in conjunction with improved fabrication methods has led to the development of LDMs for new applications, including biomedical^19,20^ and antipathogenic technologies^15,21^. LDMs that have demonstrated antimicrobial activity across a range of dimensions include 0D black phosphorous (BP)^21^; 1D zinc oxide (ZnO)^22^ and 2D molybdenum disulphide (MoS_2_)^23^; among others^13,24,25^. The antimicrobial mechanisms of LDMs have not been clearly elucidated, and there remain conflicting opinions in the field, in part due to varied experimental designs and multifaceted antimicrobial mechanisms^26,27^. It is thought, however, that the antimicrobial activity can occur through both physical and chemical means, or in combination. Importantly, it has been suggested that LDMs can limit the potential development of microbial resistance, while also enabling control over the antipathogenic mechanisms through tuning the physico-chemical properties of the materials, such as size, shape and composition, including heterostructures and the addition of functional groups^13,28^.

This review focuses on the current understanding of the antimicrobial mechanisms for LDMs and highlights areas which require further investigation. Initially, the antimicrobial mechanisms, including passive and stimuli-activated actions of LDMs, will be summarised. Following this, elemental and compound LDMs will be reviewed, focusing on graphene and graphene analogues, metal oxides (MOs), transition metal dichalcogenides (TMDs) and early transition metal carbides, nitrides and carbonitrides (MXenes) classes of materials. Finally, recent advancements in combined LDMs either as heterostructures or composite forms and their tunability in terms of antimicrobial activity will be analysed. The review concludes with a discussion of future design strategies for single and composite LDMs to optimise antimicrobial efficacy for next-generation treatments.

## LDMs—classifications and types

**Fig. 1:**
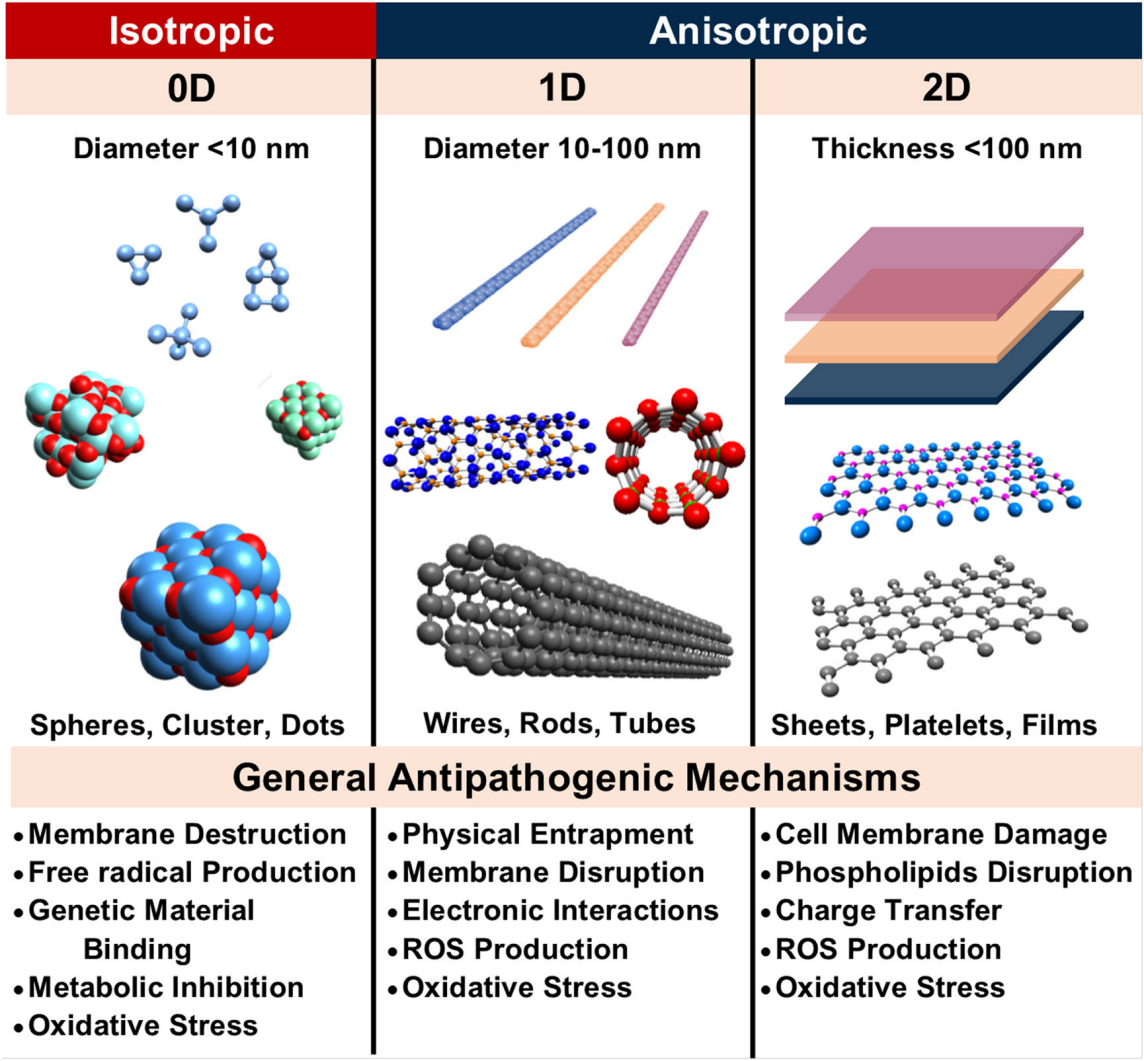
The contrast of 0D, 1D and 2D materials. Common structures for each dimensional classification and their general antimicrobial mechanisms.

**Fig. 2:**
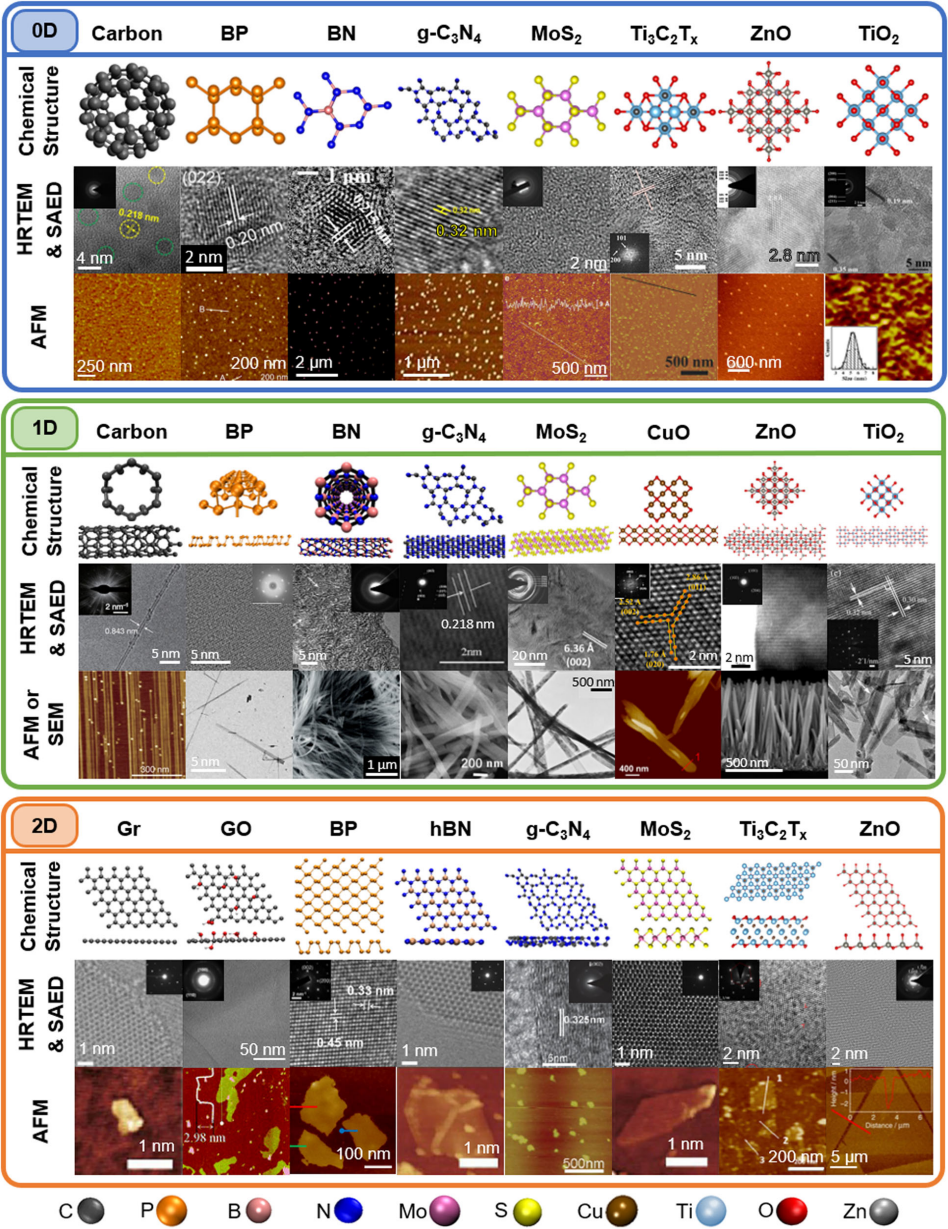
Visual summary of common LDMs. **0D** The chemical structure (top), high-resolution transmission electron microscope (HRTEM) image (inset is corresponding selected area electron diffraction (SAED) pattern where applicable) (middle) and atomic force microscopy (AFM) scan (bottom) for carbon dots^77,200^, BP QDs^201^, BN QDs^202^, g-C3N4 QDs^123^, MoS_2_ NDs^168^, Ti_3_C_2_T_x_ QDs^83^, ZnO QDs^133,203^ and TiO_2_ QDs^129,204^. **1D** The chemical structure (top), HRTEM image (inset is corresponding SAED pattern) (middle) and AFM scan or scanning electron microscope (SEM) image (bottom) for carbon NWs^205^, BP NWs^18^, BN NFs^206^/NTs^207^, g-C_3_N_4_ NRs^179,208^, MoS_2_ NWs^91,209^, CuO NRs^86^, ZnO NWs^22,210^ and TiO_2_ NWs^12^/NTs^211^. **2D** The chemical structure (top), HRTEM image (inset is corresponding SAED pattern) (middle) and AFM scan (bottom) for graphene (Gr) NSs^192^, GO NSs^212,213^, BP NSs^194^, hBN NSs^192^, g-C_3_N_4_ NSs^214^, MoS_2_ NSs^192^, Ti_3_C_2_T_x_ NSs^43,215^ and ZnO NSs^216^.

LDMs can be classified as 0D, 1D, or 2D depending on their size and aspect ratios^29^. A visual summary of LDMs and their associated antimicrobial mechanisms are shown in Fig. 1. Some properties of common 0D, 1D and 2D materials are shown in Table 1 and their atomic structure and morphology are shown in Fig. 2. Common LDMs include carbon, graphene (Gr), graphene oxide (GO), graphitic carbon nitride (g-C_3_N_4_), BP, Boron nitride (BN), 2D hexagonal BN (hBN), MoS_2_, titanium carbide (Ti_3_C_2_T_x_) MXene and MO such as ZnO, titanium oxide (TiO_2_) and copper oxide (CuO).

**Table 1:** Properties of common LDMs.

		Stable in water^a^	Stable in air^a^	Bandgap (eV)	Approximate zeta potential (mV)	Antibacterial activity
**0D**	**Carbon**	×^b77^	✓^157^	1.5^77^	−20^32,77^	✓^32^
	**BP**	×^b126^	×^158^	0.5–1.1^158^	−10^159^	✓^21^
	**BN**	✓^82^	✓^157^	6.5^160^	−50^161^	NA
	**g-C**_**3**_**N**_**4**_	✓^162^	✓^163^	2.7^164^	−40^165^	✓^123^
	**MoS**_**2**_	✓^166^	×^167^	3.9–4.2^157^	−20^168^	✓^74^
	**Ti**_**3**_**C**_**2**_**T**_**x**_	×^169^	×^169^	>0.1^83^	−60^170^	NA
	**ZnO**	✓^79^	✓^171^	3.4^13^	−40^79^	✓^78^
	**TiO**_**2**_	✓^172^	✓^172^	3.2^129^	N/A	✓^136^
**1D**	**Carbon**	✓^173^	✓^173^	0.5^174^	−30^175^	✓^56^
	**BP**	NA	✓^18^	2.6^176^	NA	NA
	**BN**	✓^177^	✓^177^	4.5^174^	−10^178^	✓^90^
	**g-C**_**3**_**N**_**4**_	✓^163^	✓^163^	2.8^179^	−30^180^	NA
	**MoS**_**2**_	✓^181^	✓^182^	2.5^183^	NA	NA
	**CuO**	✓^39^	✓^39^	3.2^184^	−20^19^	✓^39^
	**ZnO**	✓^22^	✓^135^	3.3^13^	−15^185^	✓^109^
	**TiO**_**2**_	✓^186^	✓^187^	2.5^36^	−30^188^	✓^12^
**2D**	**Gr**	×^189^	✓^190^	0^191^	−40^192^	×^[b154^
	**GO**	✓^190^	✓^190^	2.2^193^	−30^23^	✓^97^
	**BP**	×^130^	×^194^	2.0^130^	−30^45^	✓^45^
	**hBN**	×^195^	✓^20^	5.7^33^	−35^192^	✓^16^
	**g-C**_**3**_**N**_**4**_	✓^163^	✓^163^	2.1^163^	−45^196^	✓^99^
	**MoS**_**2**_	✓^49^	×^197^	1.8^198^	−35^192^	✓^47^
	**Ti**_**3**_**C**_**2**_**T**_**x**_	×^169^	×^169^	0.1–2^147^	−40^23^	✓^118^
	**ZnO**	✓^94^	✓^94^	3.4^13^	−20^199^	✓^94^

^a^Stable over 7 days.

^b^Requires functionalization.

### 0D materials

Zero-dimensional materials (0D materials) are confined to the nanoscale in all three dimensions and are composed of only a few atoms, resulting in an average size of less than 10 nm and uniform (i.e. isotropic) properties^30^. Quantum dots (QDs) are crystalline clusters of semiconductor materials^31^, while nanoclusters and nanodots (NDs) consist of a wider range of materials under 10 nm^30,32^. 0D materials have gained popularity due to their versatile size-dependent optical and physicochemical properties^14,30^, along with the possibility for biomedical applications, such as bioimaging^33,34^. Several advantages include tuneable oxidation potential^35^; light-induced free radical generation^30^; and high photothermal efficiency^14^.

### 1D materials

One-dimensional (1D) materials are confined to the nanoscale in two dimensions while the remaining dimension is typically in the microscale. These materials form cylindrical structures with a diameter ranging from 10 to 100 nm and generally are less than 12 µm in length^12,15^. Nanorods (NRo) are characterised as solid structures with a low length to width aspect ratio in comparison to nanowires (NWs), nanotubes (NTs), nanoribbons (NRi) and nanofibers (NFs), which have a high length to width aspect ratios^36,37^.  As the terms NWs and NFs are often used interchangeably, in this review, we classify NWs as structures composed of inorganic or metallic materials, whereas we define NFs as composed of organic materials^36,38^. NTs possess a hollow centre, typically with an internal diameter of under 100 nm and an external diameter up to 1 µm, which results in the highest surface area for 1D materials^37^. It should be noted that these NT dimensions are not absolute, and examples of larger materials being referred to at NTs in the literature are common. Unlike 0D materials, 1D materials are anisotropic as many of their physicochemical properties are non-uniformly distributed. Some favourable properties of 1D materials, include a larger/higher relative surface area, and tuneable oxidative stress and catalytic ability^22,36,39^. Within biological systems, 1D materials have been investigated for potential anticancer applications^40^ and other biomedical application^41,42^.

### 2D materials

Two-dimensional (2D) materials are only confined to the nanoscale in one dimension, and the other two dimensions typically span into the micro to centimetre scale^43,44^. They form nanosheets (NSs), also called nanoflakes, which can have more random shapes^45^. When materials have a more uniform and controlled configuration with straight edges, they are called nanoplatelets (NPs), or nanodisks if they have rounded edges^20^. Both NSs and NPs have a thickness under 3 nm (1–2 atomic layers)^16,45^. Like 1D materials, 2D materials are also anisotropic, with tuneable properties, such as photothermal behaviour^46^, hydrophobicity^47^ and stability in a range of chemical environments^48,49^, allowing 2D materials to be widely investigated for biomedical applications. Few-layered materials are formed when multiple atomic layers are stacked, and generally confined to a thickness of 3–50 nm thick, while their lateral area is typically under 1 cm^2 ^^27,49^.

## Mechanisms of antimicrobial activity for LDMs

The antimicrobial mechanism of LDMs is multifaceted, with the precise mechanism of action varying between materials, systems and applications. This has important implications for the design, application and deployment of antimicrobial LDMs, and their use as viable treatments. To this end, it is important to classify the various modes of action to unlock their full potential as antimicrobial agents. Further, LDMs can be non-specific, meaning that potential synergistic or antagonistic effects must be assessed.

**Fig. 3:**
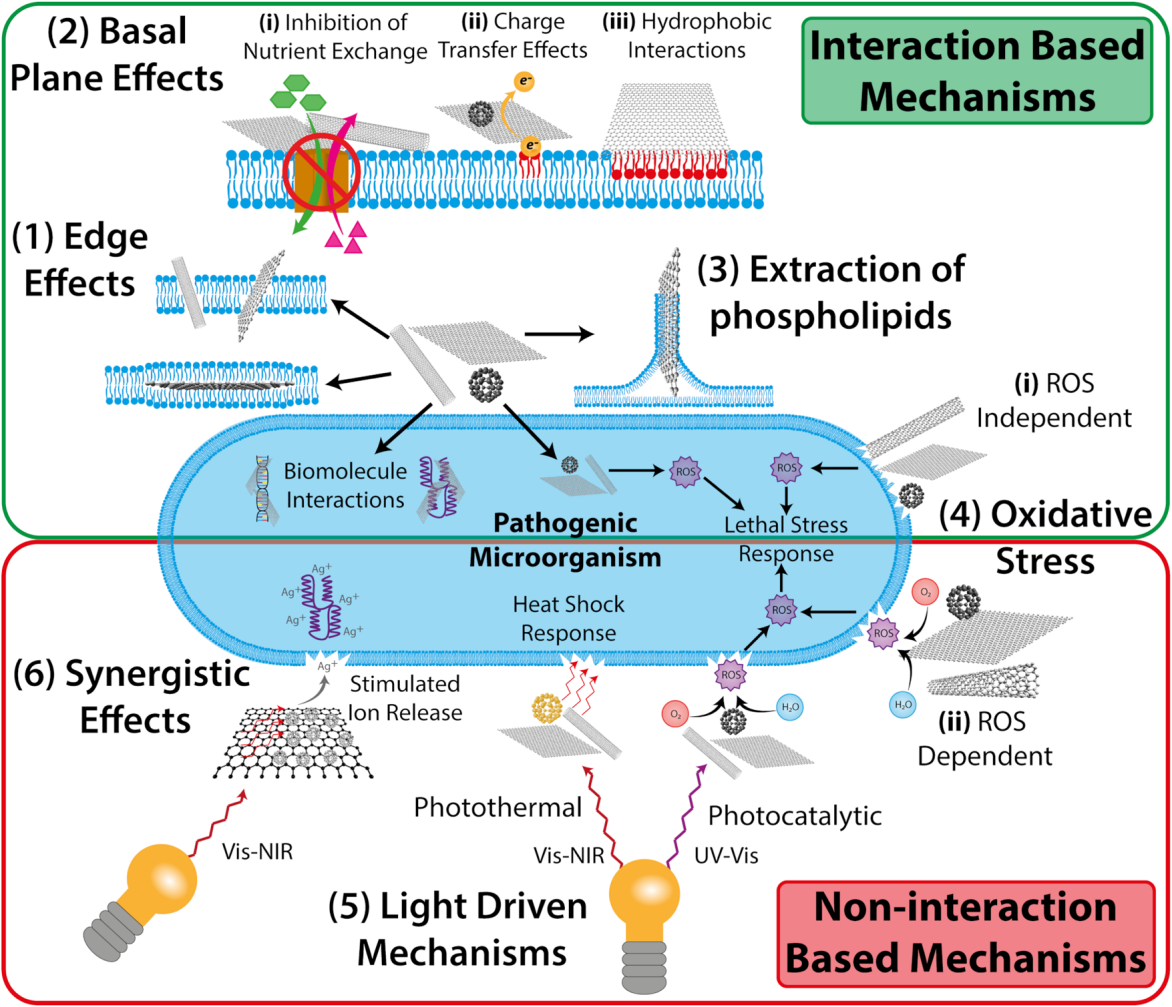
Summary of the most accepted antimicrobial mechanisms of LDMs. A more detailed explanation of the mechanisms provided in antimicrobial mechanism section.

Within existing literature, there is contention surrounding the antimicrobial mechanisms of LDMs. This is due to the complexity of the system as a whole, with interrelated and often simultaneous modes of action, the infancy of the field, and current experimental limitations often causing confusion^28^. As such, several mechanisms have been proposed, supported experimentally and theoretically, with both positive and negative correlations often being described. For clarity, we have assessed the currently reported LDM antimicrobial mechanisms and separated them into seven broad categories (Fig. 3).

1. *Basal plane effects*: Possessing a large surface area and amenability to chemical functionalisation, the basal plane of LDMs is believed to contribute to their antimicrobial activity. 2D materials have been observed to “wrap” microbial cells^50–52^, which is thought to inhibit nutrient/waste exchange^52^ and/or cause perturbations in the integrity of the cell membrane through hydrophobic interactions^51,53^, or charge transfer effects^54^; all cases resulting in cell lysis. The basal planes of 2D materials can also interact with intracellular lipids, proteins and nucleic acids, through π−π stacking, hydrogen bonding and electrostatic adsorption^28^. 0D^55^ and 1D^56,57^ nanomaterials have also been observed to demonstrate antimicrobial activity resulting from interactions with the basal plane.
2. *Edge effects:* The edge of LDMs possess unique physicochemical properties when compared to their respective bulk materials, which contribute to their antimicrobial action. Both 1D^56,58^ and 2D^23,50^ materials have been shown to puncture cell walls of microbial species upon contact, resulting in the leakage of intracellular components, and in-turn cell lysis. Experimental and theoretical studies demonstrate that 2D materials can translocate through the membrane in an orthogonal mode of penetration^59,60^, or form membrane-nanosheet sandwich structures^59,61^, due to different physico-chemical properties such as size and the degree of oxidation. These effects are not targeted and therefore, can pose a threat to mammalian cells.
3. *Extraction of phospholipids from the membrane:* Following an initial interaction with the edge of 2D materials, “nanoscale de-wetting” can occur due to strong hydrophobic interactions between the basal plane and the phospholipids, resulting in eventual membrane collapse^16,62,63^. Interestingly, 1D materials have been shown to exhibit similar phenomena on membrane models^64^ however, studies on microbial cells have yet to be conducted.
4. *Oxidative stress:* Reactive oxygen species (ROS) can cause damage to important cellular components including proteins, nucleic acids and phospholipids, resulting in cell lysis^65^. ROS-independent oxidative stress can occur naturally in microbes, and interaction with or internalisation of LDMs can dramatically increase the intracellular ROS^66^. Conversely, LDMs can generate ROS in an aqueous media, which then interacts with the microbial cells^25,67^. For LDMs, the typically generated ROS species include singlet oxygen (^1^O_2_), superoxide anion radicals (^•^O_2_^–^), hydroxyl radicals (^•^OH) and hydrogen peroxide (H_2_O_2_). A summary of redox potentials for the generation of these ROS at physiological pH are shown in Supplementary Table 1 and referenced in Fig. 3.
5. *Light-driven mechanisms:* This includes photocatalytic activity^31,68^, in which light, typically at a wavelength from ultraviolet (UV) to visible (Vis), drives the production of ROS which causes cell lysis. Alternatively, photothermal activity can be generated^25,46^, typically by light in the Vis-near infrared (NIR) wavelength region, which can cause a localised temperature increase which inactivates the microbial cells through membrane damage and the denaturation of enzymes^69^.
6. *Synergistic effect:* Combing LDMs can alter properties of materials, such as improved stability or enhanced antimicrobial activity^45,70^. One way the antimicrobial activity can be improved is in the form of releasing of ions or molecular species, also known as degradative effects^70^. Altering the materials properties, such as zeta potential, can promote new antimicrobial mechanisms^21,45^.
7. *Other physical interactions:* When 1D and 2D materials come into contact with microbial cells, they are able to physically damage the membrane^23,71^. This can be in the form of wrapping around, encapsulation or entrapping the cell, inducing cell lysis^56^. This is distinct from edge effects, which rely on either the electrical interaction of LDMs or physical damage, which occurs at the apex of an atomically sharp material.
  While these categories broadly cover the reported antimicrobial mechanisms of LDMs, these mechanisms often work simultaneously, and the relative importance of each is still debated. Broadly, the precise antimicrobial efficacy is likely to depend on: (1) biological factors, such as cell type, size and shape, (2) nanomaterial factors, such as size, shape and chemical functionality and (3) environmental factors, including, temperature, pH and components in the media such as proteins. Additionally, LDMs based nanocomposites have been developed which demonstrate multifaceted antimicrobial mechanisms and with enhanced treatment properties^6,70^.

## Fabrication of LDMs

The fabrication methodology of LDMs can also be used to manipulate their edges and basal planes. In general, synthesis of LDMs can be placed into two main categories: (1) bottom-up or (2) top-down methodologies. Bottom-up approaches are characterised by growing/synthesis from chemical precursors using a range of procedures and are more commonly used for metal oxides and 1D structures^72,73^. Alternatively, top-down synthesis occurs when a bulk material is broken down into the desired nanostructure and are typically used for 0D and 2D materials^25,74^.

## Suspension-based LDMs as antimicrobial agents

**Fig. 4:**
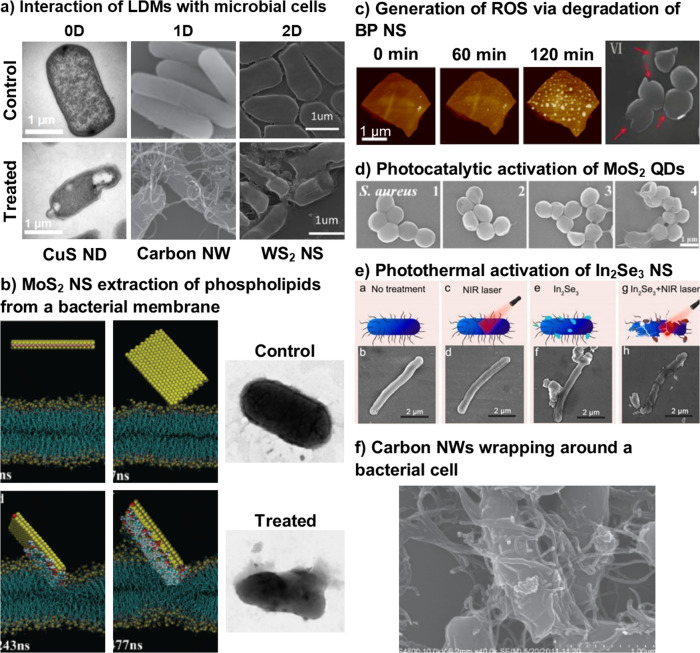
Summary of antimicrobial interactions of LDMs in suspensions. **a** Interaction of suspended Copper sulphide (CuS) NDs with *Escherichia coli (E. coli)*^75^, carbon NTs wrapping around *Lactobacillus acidophillus (L. acidophillus*)^56^ and tungsten disulphide (WS_2_) NSs with *E*. coli^106^. **b** The extraction mechanism of membrane phospholipids by MoS_2_ NSs^63^. **c** Generation of ROS via degradation of BP NSs over 120 min (left)^217^ and the interaction of Ti/BP NSs film with *S. aureus* (right) with cellular damage indicated with red arrows^6^. **d** Photocatalytic activation of MoS_2_ QDs against S*taphylococcus aureus* (*S. aureus)*^74^ and **e** the photothermal activation of indium(III) selenide (In_2_Se_3_) NSs against *E. coli*^46^. **f** Carbon NWs wrapping around *L. acidophillus*^56^.

An effective and common method of utilising the antimicrobial properties of LDMs are as suspension-based approaches. LDMs can be suspended within a range of liquid medium and allow for the free movement of LDMs along with providing a possible environment for the generation of ROS. This section will highlight example LDMs in suspension with antimicrobial properties. Figure 4 shows common interactions of selected suspension-based LDMs and microbial cells to provide an overview for the reader.

### 0D materials

Suspension-based 0D materials have several reported antimicrobial mechanisms, including cellular uptake^75^, radiation stimulus^74^ and ROS generation^31^. Due to their innately small size, 0D materials can easily transverse microbial cell membranes, and enter the intracellular space (Fig. 4a). This provides a unique capability to 0D materials, which is often not observed for 1D and 2D materials due to size constraints. Once inside the pathogenic cell, 0D materials can disrupt internal cellular processes (metabolic process, deoxyribonucleic acid (DNA) transcription, respiration, etc.) and also damage internal cellular material (DNA, ribosomes, and plasmids, etc.)^76^. The precise mechanism is dependent on the specific LDM, but 0D materials can rapidly lead to cell lysis following cellular uptake.

Further, 0D materials have also shown potential for photo-induced antibacterial treatments (Fig. 4d, e). Semiconductor QDs with a bandgap greater than 3.1 eV have demonstrated non-specific toxicity towards both mammalian and microbial cells when illuminated with UV light^72^. However, other semiconductor QDs are typically less toxic towards mammalian cells, hence more biocompatible, when excited under visible or NIR light^21^. Regarding the microbial toxicity, materials including carbon NDs (CDs)^77^, BP QDs^21^, cadmium telluride (CdTe) QDs^35^, MOs QDs^75^ and MoS_2_ QDs^74^, can generate excess ROS under visible, NIR and UV light irradiation. Metal oxides, such as ZnO QDs^78,79^ and vanadium oxide (Vo*x*) QDs^80,81^ can also release ions under UV and ambient light irradiation. Tailoring the dimensions tunes the electronic structure of these LDMs to manipulate the wavelengths of light that activate the particles. Bare copper sulphide (CuS) QDs have three reported mechanisms, photothermal effects, Cu^2+^ ion release and photodynamic generation of ROS under NIR^75^. These examples have demonstrated the importance of light in the antimicrobial mechanism of 0D materials, indicating the potential use in photodynamic therapy. Conjugating 0D materials with other LDMs can synergistically enhance the antibacterial mechanisms, by enhanced ROS production^78^ or light assisted drug delivery^21^.

Research into 0D materials as antimicrobial agents is relatively recent, and several materials have potentially favourable properties. BP QDs have recently been used for bioimaging and drug delivery applications, demonstrating biocompatiblility^82^. Larger TiO_2_ NPs have demonstrated ROS generation, but research into QDs is lagging. While MXene QDs, including titanium nitride (Ti_2_N), Niobium carbide (Nb_2_C) and Ti_2_C_3_T_x_, have shown photothermal properties, with possible uses in cancer treatments and bioimaging^83^, to the best of our knowledge, no tests have been so far conducted into their potential use as antimicrobials. Further, bare WS_2_ QDs had no antimicrobial impact, but when conjugated with antimicrobial peptides, it was effective against both *Pseudomonas aeruginosa (P. aeruginosa*) and *C. albicans* biofilms^84^. This highlights the potential for more 0D materials to potentially have antimicrobial properties, or to have induced action through conjugation, but further exploration into these materials is needed.

### 1D materials

Similarly, research into antimicrobial 1D structures, including carbon NTs^56^ and a range of MOs^13,29^, is increasing. There are different antimicrobial mechanisms that have emerged in 1D materials (Fig. 4a), such as carbon NTs, deactivate microbial cells through physical damage, which is heavily influenced by size^71^. For thinner single-walled NTs (SWNTs), the main mechanism is to puncture the bacterial membrane, acting as a nanodart/nanoknife^71^. Thicker, multi-walled NTs (MWNTs) tend to be less effective^71^ and predominantly wrap around bacteria^56^ (Fig. 4f). Both SWNTs and MWNTs are potentially toxic against a range of microbes found in the human gut, including *E. coli, S. aureus* and *Enterococcus faecalis (E. faecalis)*^56^. Alternatively, MOs NRs and NWs mainly generate ROS to chemically induce cell lysis^85^. Interest into MOs has mainly focussed on ZnO^85^ and CuO^86^, however recently other MOs, such as cerium(IV) oxide (CeO_2_)^29^, magnesium oxide (MgO)^87^, maghemite (Fe_2_O_3_)^88^ and nickel(II) oxide (NiO)^40^, have demonstrated promising antimicrobial properties. ZnO materials can also release Zn^2+^ ions, along with ROS, to aid in the damage of microbial cells^85^, indicating the possibility that LDMs with non-physical modes of action could have antimicrobial potential.

There are several materials that have been shown to possess antimicrobial properties in their 2D form, but there is little research into their corresponding 1D forms. One material with such potential is graphitic carbon nitride (g-C_3_N_4_), which generates ROS under light irradiation^89^. Recently, polymer functionalised BN NTs were also shown to have an increased efficacy compared to pristine BN NTs^90^. This further indicated how chemical modifications of 1D materials can enhance the antimicrobial activity to materials with little-to-no inherent efficiency. Other 1D materials have attracted interest for applications including gas sensing^18^, catalysis^91^ and biomedicine^92^. Recent advancements in fabrication techniques have led to the synthesis of materials like BP NWs and NRis^18,93^, which to our knowledge have not been explored for their antimicrobial properties.

### 2D materials

Recently, 2D materials have received considerable attention due to their relative ease of fabrication and their amenability to tuning their properties to make them responsive to a wider range of stimuli^45,48,94^. There are several common antimicrobial mechanisms observed in 2D materials, including physically slicing through the membrane^49^ (Fig. 4a), phospholipid extraction^62^ (Fig. 4b) or generating reactive oxide species^70^ (Fig. 4c).

#### Graphene analogues

Pure graphene has limited antimicrobial properties, while its oxide derivatives GO and reduced graphene oxide (rGO) have demonstrated broader antimicrobial capabilities^62,95^. Both GO and rGO can deactivate bacterial cells via oxidative stress^96^ and piercing the membrane^53^, along with potential destructive extraction of the phospholipids comprising the membranes^62^. When GO was incorporated into an injectable hydrogel, its high photothermal properties and increased conductivity lead to improved antimicrobial activity against *E. coli* and MRSA^95^. It should be noted that the antimicrobial activity of graphene, GO and rGO remains controversial. A recent paper suggested that pure GO possess no inherent antibacterial character, but rather the widely reported behaviour is due to impurities^97^.

Other graphene analogues also display antimicrobial properties. BP and hBN NFs have demonstrated the previously mentioned common antimicrobial mechanisms against *E. coli* and *S. aureus*^25,45^. Similar to GO, it has been speculated that hBN NSs cause membrane stress through lipid extraction^16^. BP NSs generate ROS under NIR light irradiation^25^, which can be enhanced when combined with larger nanoparticles^70^.

#### Graphitic carbon nitride

Another 2D carbon-based material with antimicrobial potential is g-C_3_N_4_. Pristine g-C_3_N_4_ can effectively be used to treat against *E. coli*, MRSA and *Bacillus anthracis* spores, under visible or UV light irradiation conditions^98^. The functionalization of the g-C_3_N_4_ surface using nitrogen plasma treatment (N-g-C_3_N_4_) can increase the activity without requiring light activation^99^. The primary antimicrobial mechanism of pristine g-C_3_N_4_ is via the photoactivated generation of free radicals^98^, while N-g-C_3_N_4_ is thought to induce cell death mainly through the interaction with phospholipids within the cell membrane^99^.

#### Metal oxides

2D MOs such as ZnO^94^, TiO_2_^12^, CuO^100^ and MgO^27^ have gained attention for effectiveness against a wide range of pathogenic microbes. The main antimicrobial mechanism used by MOs is the photo-induced generation of ROS^100,101^. Under visible light, MOs are effective treatments against *S. aureus*, *E. coli* and *Staphylococcus epidermidis* (*S. epidermidis)*^27,102^, as well as under UV light^94^. Some MOs including ZnO and CuO release cations that electrostatically disrupt the membrane^102^.

#### Transition metal dichalcogenides

TMDs also generate ROS and damage cellular membranes^49^. Recent studies have focused primarily on MoS_2_ and WS_2_ as potential antimicrobials as they are non-toxic^103^. The antimicrobial mechanism for MoS_2_ is multifaceted, with the primary mechanism involving the generation of superoxide anions^67^, combined with slicing the membrane^49^ and binding to peptide backbones^104^. Alternatively, WS_2_ primarily deactivates cell through membrane damage, not through ROS^105,106^. The potential antimicrobial properties of other TMDs remain to be explored.

#### Other 2D materials

Several other 2D materials have shown potential for antimicrobial applications in limited studies but require further research. Preliminary studies into MXenes NSs, mainly Ti_3_C_2_T_x_, and indium(III) selenide (In_2_Se_3_) have demonstrated activity against a limited number of Gram-positive and Gram-negative bacteria^24,46,50^. The sharp edges of the NSs play a key role in the degradation of the cellular membrane^24^. The photothermal properties of In_2_Se_3_ can also be used to increase the antimicrobial efficacy^46^.

As new LDMs are developed for use within other applications such as electronics or sensing, their potential as possible antimicrobial agents should be explored. Enhancing the antimicrobial capabilities of LDMs though heterostructuring will be discussed in a later section.

## Surface coated LDMs as antimicrobials

### Planar surfaces

**Fig. 5:**
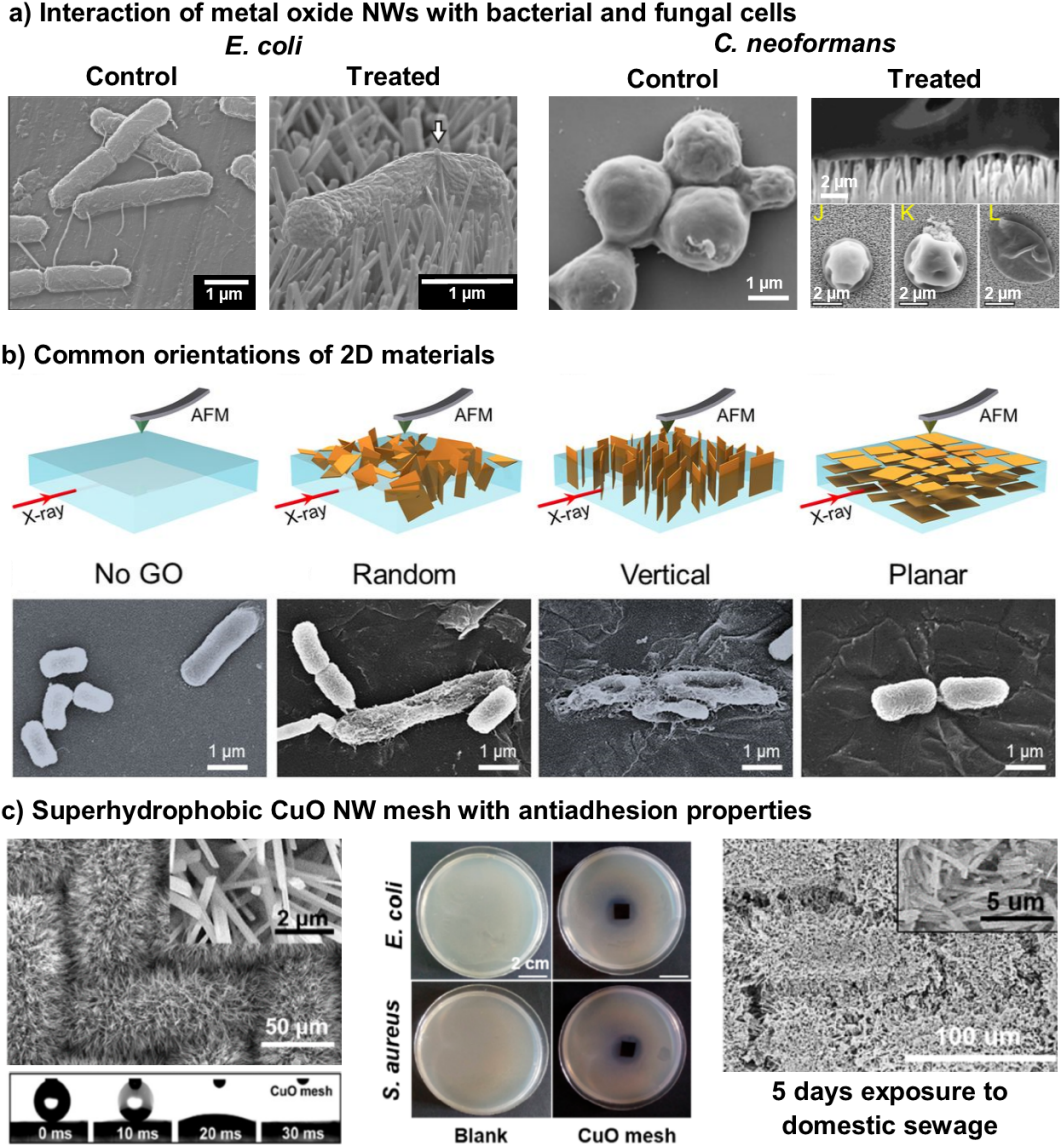
Summary of antimicrobial interactions with surfaces modified with LDMs. **a** Membrane damage of *E. coli* from TiO_2_ NWs^8^ and *Cryptococcus neoformans* from ZnO NWs (top and side view)^22^. **b** Various orientations of GO NSs on a surface and their interaction with *E. coli*^110^. **c** A multi-functional CuO NWs mesh with a superhydrophobic surface (left) which prevent *E. coli* and *S. aureus* growth (middle) and microbial adhesion after 5 days of incubation in domestic sewage water (right)^39^.

LDMs can be used to either generate or boost antimicrobial activity of a surface. For 1D and 2D materials, the orientation of the LDMs can influence the antimicrobial properties and affect the action of the primary antimicrobial mechanism (Fig. 5). The main orientations are vertical arrays^23^, horizontal coatings^107^ or randomly oriented arrays^26^. These arrays and coatings can be used for implants^41^, wound dressings^15^, and other medically relevant surfaces^12^.

Vertical arrays of LDMs are often reported for antimicrobial capabilities, through chemical activity and improved membrane damage, derived from their sharp edges (Fig. 5a, b)^12,108^. 1D MOs have gained substantial attention as surface modifiers recently, with studies highlighting ZnO^22^, TiO_2_^12^ and CuO^108^ arrays as potential antibacterial and antibiofilm surfaces, as well as a possible antifungal treatment^22^. When MOs are grown in vertical arrays, there is a synergistic effect between the physical puncturing of the cellular membrane, the generation of ROS^22,108^, and the electrostatic interaction of the positive metal ions and the negatively charged membrane^109^. Combining 2D and 1D structures in an array can enhance photoactivated antibacterial properties by altering the electronic band structures of the materials. When rGO NSs are combined with CuO NWs, the CuO injects an electron into the rGO, allowing for enhanced ROS generation under visible light irradiation^108^. A recent study found that a higher edge density of an array can improve the antimicrobial activity, whilst also supressing any mechanisms of the substrate, such as wrapping^23^. The morphology of the LDMs in the vertical array influences the antimicrobial efficacy; however, more research is needed to identify they key parameters for optimisation.

Similarly, randomly orientated LDMs (Fig. 5b) are used to enhance the antimicrobial properties of a surface. These materials are still able to penetrate cellular membranes through edge effects; however, the number of edges in the preferential orientation is reduced^26,110^. Randomly orientated GO NSs effectively reduced a population of *E. coli* by 25%, compared to 44% for vertically aligned NSs^110^. Although the main mechanism for GO NSs is still being investigated, the decreased efficacy of random NSs was attributed to the decreased penetration of the cellular membrane^110^. Randomly orientated 1D MOs NWs can influence the hydrophobicity of the surface, leading to a two-stage mechanism (Fig. 5c). The more tightly packed surfaces increase the hydrophobicity, preventing cellular adhesion. Once cells begin to settle, the NWs can puncture the membrane, whilst the chemical activity of the MOs NWs also provide longer-term antimicrobial action^26^. Initial research has been conducted into the relationship between material angle, microbial adhesion and membrane penetration. As fabrication methods continue to advance to allow for greater control of the angle, this relationship can be explored in more detail to allow for greater optimisation.

Although still capable of antimicrobial action, horizontal or planar LDMs (Fig. 5b) are not as effective as the previously mentioned orientations. When deposited horizontally, LDMs are more reliant on chemical mechanisms, as the physical antimicrobial mechanisms derived from edge effects are limited^15,110^. For 1D MOs deposited horizontally, electrostatic interactions of ROS are predominantly used to inactivate cells^15,41^. In the case of CeO_2_ NRos deposited onto medical-grade titanium, the material was able to reduce biofilm formation of common plaque-forming bacteria by 99%. The main antimicrobial mechanism is believed to be via electrostatic interactions between the CeO_2_ NRos and the cell membrane^41^. Horizontally orientated TiO_2_ NWs coatings rely on ROS, as the NWs are more efficient at photodegradation than larger nanoparticles^15^. Horizontally deposited NSs tend to show lower antimicrobial activity compared to vertically orientated NSs^107,110^. For example, horizontal GO NSs resulted in a ~20% reduction of *E. coli* cells, in comparison to 44% for the vertical GO NSs^110^. MoS_2_ NSs have been shown to reduce *E. coli* growth by roughly 50% after a 3 hour incubation^107^.

Surfaces can also be modified with the addition of 0D materials to improve their antimicrobial properties. The antimicrobial activity of 0D material modified surfaces has been investigated across a broad range of medically relevant materials, such as wound dressings^111,112^, resins^113^, polymers^114^ and drug delivery systems^115^. Due to the shape of 0D materials, chemical pathways such as ROS generation^112^ or electrostatic interactions^115^ are the main antimicrobial mechanism. Depositing carbon and graphene QDs onto common fabric bandages prevent *E. coli* and *S. aureus* growth and promoted wound healing via photo-induced ROS^112^. When carbon QDs were doped with nitrogen, they were capable of treating MRSA infections to the same degree as the antibacterial drug vancomycin^111^, indicating its potential use in treating vancomycin-resistant *Enterococcus* infections. Biofilm formation of *Streptococcus mutans*, a common dental pathogen, had a 99% reduction after exposure to an adhesive dental resin coated with ZnO QDs^113^. Further, *E. coli* and *S. aureus* growth was reduced by ZnO QDs deposited onto bioactive glass nanoparticles^79^. The antimicrobial action of 0D materials can be increased by depositing multiple materials that have a combined effect. The ROS generation of ZnO can be increased by also depositing cadmium sulphide (CdS) QDs onto the surface and activating using UV light. Combining both QDs with the polysaccharide chitosan prevented both *E. coli* and *Bacillus subtilis* (*B. subtilis)* growth^115^. Using 0D materials as additives in materials with inherent antimicrobial properties can also increase the effectiveness. Under ambient light, indium-based QDs were not effective against clinical and environmental strains of *P. aeruginosa* and *E. coli*. Combing the indium-based QDs with crystal violet increases light absorbance and increases ROS generation to a lethal amount^114^.

### Three-dimensional filters

LDMs can be deployed to improve water filtration to prevent infections from waterborne pathogens. One method to increase the efficacy of water filtration is to increase the hydrophilicity of the filter. This can be achieved by depositing 2D materials such as WS_2_^48^ or g-C_2_N_4_^116,117^, or growing 1D materials like Cu NWs^39^ (Fig. 5c). WS_2_ was able to reduce the amount of *E. coli* and *S. aureus* in solution by around 90%^48^. Photocatalytic materials such as g-C_2_N_4_ NSs also generate ROS under visible light exposure and have been shown to cause an over 99.99% reduction *E. coli* from solution^117^. Cu NW mesh uses multiple antimicrobial mechanisms, including super hydrophilicity, the generation of Cu^+^ ions and photothermal interactions^39^. MXene membranes have also demonstrated antifouling properties and were effective against both *E. coli* and *B. subtilis* via oxidation and photothermal reactions^118^.

Another application for LDMs is enhanced filters for use in air purification to reduce the spread of airborne pathogens. Fe_2_O_3_ NWs grown on an iron mesh to capture common indoor bioaerosols via the generation of hydroxyl radicals^73^. A single filter was able to capture 52% of airborne *S. epidermidis* and *E. coli*, and 5 filters in tandem were able to reduce airborne cells by 98%. Although a promising application, the instability of several LDMs in ambient conditions has limited their potential to be used for air purification.

## Hetero-LDMs: current research and perspectives

When dissimilar materials are stacked in layers, they can result in new properties which have typically been used to great effect in electronics and optoelectronics^119,120^. The ability to stack materials of desired size/thickness and different phases (solid/liquid) in lateral or vertical heterostructures, allows the manipulation of physical and electronic properties of the material at the atomic scale via quantum engineering^121^. The use of LDMs can be considered a win–win solution in the quest for achieving a combination of antimicrobial properties. Combining materials can aid in passivating materials from ambient degradation that otherwise show outstanding antimicrobial properties. These hybrid materials can also be prepared in a solution phase which can be used as surface/spray coatings on the implants using the dispersion technology^122^. The materials used to form heterostructures are usually chosen from the periodic table among the III-V, IV-IVI, II-VI groups. The material systems are stacked, altering their band structures to benefit the desired applications^119^ (See supplementary document).

## Design parameters for LD-based next-generation antimicrobials

Recent advancements in LDMs have highlighted their potential as antimicrobial treatments. These highly antimicrobial materials can reduce the number of microbial cells by over 80% within a few hours. Such materials include: (1) 0D^77^ and 1D^71^ carbon, (2) 0D^123^ and 2D^124^ g-C3N4, (3) 0D CdTe^35^, (4) 1D metal oxides^29,109^, (5) 2D GO^110^, (6) 2D BP^41,70^ and (7) 2D MXene^24,50^, which have all exhibited high antimicrobial ability. Most of these materials employ a combination of mechanisms that are influenced by their chemical composition and morphology^27,117^. There are many factors to consider when selecting LDMs for a specific antimicrobial treatment or scenario. For instance, is the desired application biological or abiotic in nature? What is the delivery method of the required treatment (injectable, solution, surface, physical, etc)? Does the treatment need to last for a prolonged period, or does it degrade rapidly in response to treatment? Is there a single target pathogen or more-general antimicrobial activity required? Is a chemical approach or physical methodology better for the scenario in question?

To assist with answering these questions, the following section has been designed to give an overview of the currently available antimicrobial LDMs and provide insight into the future directions of research in this area. Notably, the selection of LDMs is not trivial and is dependent on the system, application and specific microbes involved. Furthermore, the application must be adequately tailored to either prevention or treatment. Table 2 summarises the currently measured antimicrobial efficacy, the respective cytotoxicity, relative commercial costs, chemical stability, and the current status of the LDMs previously described for medical, industrial, and scientific-based antimicrobial-based exploitation.

**Table 2:** Summary of LDMs for antimicrobial applications^218–263^.

^a^At least one Gram-positive and one Gram-negative bacterial cell, and one fungal cell (no fungi tested+).

^b^More than three mammalian cell lines tested.

^c^Green is <5 h, yellow is 5–12 h, orange is 12–24 h and red is >24 h.

^d^Green is < $499, orange is $500–$999, red is >$1000 (estimated using Sigma-Aldrich).

^e^Newly fabricated.

When considering using LDMs within a biological system, the biocompatibility with mammalian cells is an essential factor. Table 2 summarises which LDMs have been tested for their effect on mammalian cells, either using cell cultures (in vitro) for 48–72 h^41,125^ or live mouse models (in vivo) for over a period of seven days^81,126^. If the material has demonstrated non-selective toxicity towards both microbial and mammalian cells, their use in treatment is not desirable.

### Utilising the chemical routes for antimicrobial activity

Many LDMs that have demonstrated high antimicrobial activity can generate ROS. The ability to generate ROS in solution is directly linked to LDMs bandgaps and therefore redox potentials, with the bandgaps of numerous LDMs discussed above falling into the range 1.5–3.5 eV (see Table 1). These bandgaps are sufficient to elicit the generation of ROS (Supplementary Table 1) in solution, which can damage the microbial cell wall. Therefore, if the antimicrobial action requires the production of ROS, the use of materials which fall outside of this range is not recommended. LDMs that have demonstrated antimicrobial activity and are capable of generating ROS species in solution include BP^70^, MXene^118^ and WS_2_^103^. However, there are also LDMs that do not generate ROS, but is still possess antimicrobial activity such as 2D hBN^127^, which has limited antibacterial efficiency^90,128^. For such materials, forming composites or heterostructures can alter their intrinsic bandgaps, and facilitate the generation of ROS^115,129^. In some systems, the generation of ROS can be promoted via the addition of ultra-low concentrations of hydrogen peroxide^81,112^.

LDMs which readily degrade into fragments, ions, sub-species and/or ROS can be advantageous for antimicrobial treatments or surface-functionalisation. For example, BP is well known to degrade under ambient atmosphere and solution conditions, producing ROS and P_x_ ions^130,131^. The ability for the material to both degrade and produce antimicrobial species is useful for applications which require the biocidal agent to disintegrate from the treatment zone without removal. Other LDMs degrade at a slower rate, such as In_2_Se_3_, and have also shown potential antimicrobial activity^132^. This degradation or generation of ROS can also be enhanced using light irradiation^35,124^. LDMs that have demonstrated photocatalytic properties can potentially be used to assist with targeted treatments^21^.

### Morphological and physical exploitation

The morphology, size, surface charge and flexibility of LDMs can positively and negatively correlate to biocidal enhancement in both suspension-based and surface-based technologies. Broadly, the degree of antimicrobial activity within a system is both species and treatment dependent and represents an interplay between contributing factors. However, the precise nature of the LDM-microbial interactions is still relatively poorly understood, often resulting in conflicting results, even amongst similar systems. The following section aims to collate the current level of understanding of the morphological and physicochemical interactions which facilitate LDM antimicrobial action.

For 0D materials, cellular uptake, electrostatic disruption, and specific cell–surface interactions are the primary physical modes of action. Here, the comparatively small size of the material facilitates cellular uptake, which is often not achieved by larger 1D and 2D materials. This means that the physical size and surface chemistry of the material is a key determining factor. If an application requires intracellular interactions, then 0D materials are prime antimicrobial candidates - smaller 0D LDMs can possess enhanced activity^133^. Further, 0D materials are known to facilitate intra- and extracellular damage via unfavourable electrostatic interactions. However, we found no reports of 0D LDMs that cause physical-based membrane rupture.

For 1D and 2D materials, the aspect ratio can influence the antimicrobial activity^29^. For CeO_2_ NRs, a higher aspect ratio resulted in more active sites on the NR surface, generating a higher concentration of hypobromous acid (HOBr), increasing the antimicrobial activity^29^. The aspect ratio can also be linked to the cytotoxicity of a material^29^. For some materials, a higher aspect ratio increases the toxicity towards human cells^29^. 1D CuO NW mesh was found to be superhydrophobic and prevented bacterial adhesion^39^. For 2D sheets, the edges of the material are known to be “sharp” and induce microbial cells damage upon cell-on-edge adsorption.

Several forces are at play when LDMs and microbes interact, including electrostatic, van der Waals and hydrophobic forces. Together, these forces can lead to microbial membrane damage during LDM material interactions. It should be noted that LDM-pathogen interactions are complex, possessing contributions from both the material and the microbial cell. For instance, the surface chemistry, charge, hydrophobicity, inherent roughness, geometry and free energy are all contributing factors from the LDM material. For the pathogen, molecular composition, surface charge, hydrophobicity, extracellular polymeric substance (EPS) and cell appendage interactions all play a role. In general, it has been suggested that positively charged materials attach to the negatively charged microbial membrane to induce membrane damage^45,100^. Some materials can deactivate membrane components, such as the thiol group, through the generation of ions^41,100^ or by extracting of phospholipids^62,63^. Ions are typically generated by MOs, which cause the leakage of intracellular components^39,79^ or directly damage the intracellular components^134^.

### Pre-infection treatments

One application for LDMs is in preventing microbial infections (i.e. pre-infection treatment) via limiting microbial adhesion to surfaces^26,135^ and decreasing microbial growth^32,79^. For LDMs to be used as an effective pre-infection treatment, they will need to be in a portable form with long-term stability, such as a bandage or implant coating or LDMs suspended in hydrogel^95,112^. External stimuli can be applied to prevent infections in a clinical setting but are not practical for consumer products. Similarly, the chemical stability of LDMs must be improved for commercial products as they will need to be stored for longer periods. Overall antimicrobial activity is another important property to consider when using LDMs for pre-infection treatments. Materials, such as BP NSs^45^ and g-C_3_N_4_ QDs^123^, can be used as fast-acting treatments, while other materials including 0D MOs^41,136^ and MXene NSs^137^ have shown antimicrobial activity over several days. This duration is important as it influences the frequency of treatments, and how often the wound is exposed to unsterile conditions, increasing the risk of re-infection.

### Post-infection treatments

Once an infection has formed, it can become much harder to treat and prevent more entrenched infection^138,139^. The use of stimuli-activated LDMs, such as photothermal^75^ or photoactivation^35^ could be utilised within clinical settings. Importantly, LDMs have the ability to treat established infections with large quantities of microbial cells^138,140^. One method of achieving this higher microbial inactivation can be the use of external stimuli, such as light activation^123,141^. Several materials, including 1D MOs^26,41,140^ and graphene QDs^112^ have been able to reduce pre-existing biofilms by over 90% following photoactivation. Following the initial treatment to reduce the infection, previously discussed pre-infection treatments can be used in tandem to help prevent another infection.

### Computational modelling as a guide for future treatments

**Fig. 6:**
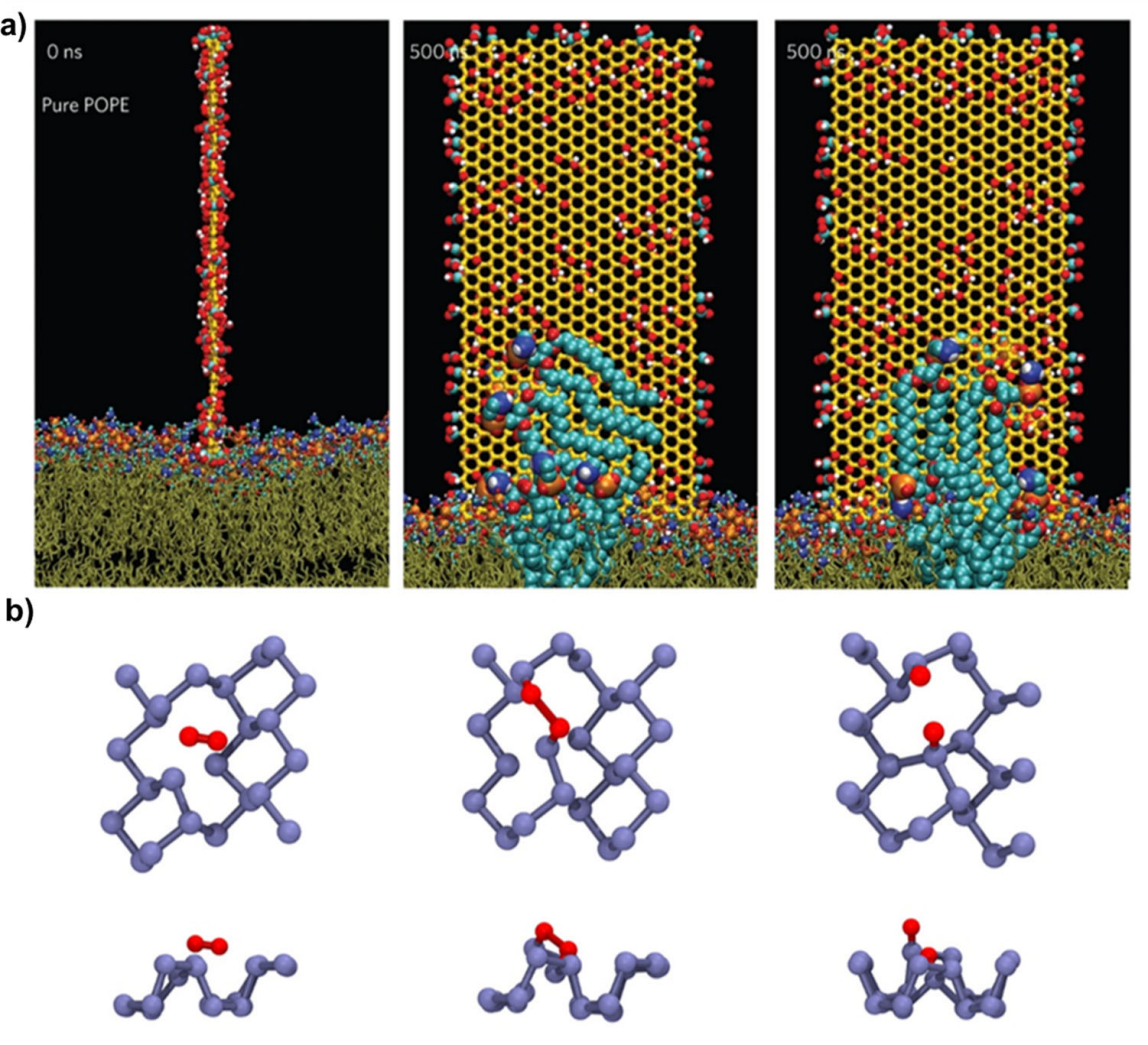
Atomistic modelling of antimicrobial LDM behaviour. **a** Lipid extraction by graphene oxide nanosheets from the outer membrane surface^62^. **b** Top view and side view of the reaction of O_2_ (red) with monovalent defect BP (purple) from QC calculations.

Computational modelling techniques have demonstrated great potential to aid in the understanding of existing antimicrobial mechanisms and guide development of new LDMs. Classical molecular dynamics (MD) simulations have been used to show in atomistic detail how GO, N-g-C_3_N_4_ and MoS_2_ nanosheets can destructively extract lipids from bacterial membranes^62,63,99^ (Fig. 6a). In addition, coarse-grained MD simulations allow for a direct and fast in silico screening of LDMs candidate materials^142^. MD simulations have also shown why some LDMs are effective in vitro but not in vivo. For example, Duan et al. demonstrated that the efficacy of GO as an antimicrobial agent was significantly reduced by the presence of a protein corona formed by serum proteins that reduced the available surface area and sterically hindered membrane penetration and disruption^143^. On the other hand, MD simulations have also shown how the effects of a protein corona can be overcome, or even utilised advantageously, for cell penetration of functionalized nanoparticles^144^. While MD simulations are useful for studying interactions that can occur between LDMs and microbial membranes or biofilms^145,146^, quantum chemical (QC) methods can calculate bandgaps of candidate LDMs^147,148^, or examine the reaction mechanisms involved in ROS generation. Taking BP as an example, while the full ROS production reaction mechanism has yet to be elucidated, studies have shown that initial reactions leading to ROS production and BP degradation are most likely to occur at edges and defects in BP^17,149^ (Fig. 6b). For the rapid and efficient exploration of a large number of candidate LDM properties, machine learning (ML) is often the best approach. ML algorithms can predict properties ranging from bandgaps of MXenes and hybrid 2D materials^150,151^ to biocompatibility of ZnO nanoparticles^152^.

### LDMs with little or no antimicrobial activity

Although many LDMs have demonstrated high antimicrobial efficiency (over 80% cell death within several hours), there are some materials with little or no antimicrobial action. These materials were only capable of killing less than 60% of the microbial cells in 3 h or required over 8 h to inactive over 70% of microbial cells. These materials include hBN NSs^153^, WS_2_ QDs^84^, BP QDs^21^ and graphene NSs^154^. Some morphologies of these materials have demonstrated higher antimicrobial efficiency. One example of decreased antimicrobial action is BP, with the 2D BP achieving over 80% cell death within an hour^25,70^, compared to QDs, which reached 60% cell death after 2 h^21^. Another example of a material with little antimicrobial activity is 2D hBN, with a 30% cell reduction in 3 h^153^. This reduced antimicrobial activity is likely due to hBN not generating ROS, which means it is more reliant on a physical rather than a chemical mechanism which limits the overall antimicrobial potential^127,153^. A careful review of the literature reveals the several QDs have lower antimicrobial activity compared to their 1D or 2D counterparts. This may be due to their antimicrobial activity arising from chemical/ion interactions^74,79^, while having diminished ability to induce membrane damage.

The medium of the LDMs can also influence the antimicrobial efficiency. MOs have an increased antimicrobial potential on a surface^12,140^, where most 0D materials are more effective as a suspension^72,77^. For LDMs that rely on chemical interaction, suspension-based approaches have a higher antimicrobial efficiency^72,140^. In contrast, physical-based antimicrobial mechanisms are efficient as both surface- and suspension-based treatments, depending on the desired application^23,116^. For water purification, membranes equipped with LDMs are more effective than LDMs freely suspended in solution, and do not have to be removed from the purified water^27,39^. For wound treatments, however, depositing LDMs onto traditional wound dressing surfaces, such as bandages or adhesive resins, have shown to promote improved wound healing compared to untreated dressings^75,112^.

## Current challenges

Although LDMs have promising antimicrobial properties, there are still limitations in fabrication processes and scalability that prevents practical implementation, for instance;

1. Synthesis methods for LDMs often use toxic solvents^87,137^, require prolonged synthesis at high temperatures^18,116^ and result in low yields^25,111^.
2. Many 1D nanostructures, aside from MOs and GO, have only recently been synthesised and currently lack exploration into possible antimicrobial activity^18,93^.
3. Some LDMs are less stable in desired environments such as in air or in solutions with a neutral pH^25,136^.

One of the significant concerns using LDMs within a medical and commercial setting are related to their fabrication. Although LDMs should be available for feasible consumer products, consistent, cost-efficient methods need to be developed to allow for batch production. Major challenges to resolve for LDMs to attain market growth include (1) scalability, especially roll-to-roll manufacturing, (2) repeatable and reliable fabrication methods, (3) low contact resistance and (4) LDM-based precise characterisation techniques. One key point to a favourable outcome is the capability to prepare 2D materials at the wafer level. In this way, it will be possible to create large amounts of 2D devices for fabrication and decrease product cost^120^. The current use of either high temperatures or toxic solvents^18,116^ has led to an increased interest in developing green synthesis routes using natural materials for LDMs fabrication^40,82^, increasing the potential for wider biomedical applications. Several current fabrication processes can take several days^15,47^, and long-term storage can be limited^24,49^.

One method of overcoming the rapid degradation of LDMs is to suspend the LDMs in liquid stabilisers or through storage in controlled environments^35,136^. For example, some materials require a specific pH for storage of more than a few weeks, which is not ideal for biomedical applications^35,72^. Although these stabilising measures are effective within a controlled laboratory setting, implementation on a larger scale for practical use is limited. For clinical applications, stabilisation could be achieved by embedding LDMs in medically relevant materials currently being used as wound treatments, such as hydrogels^155^. The scalability and long-term impacts of LDMs on biological systems still require more research. There have been some cytotoxicity studies for a range of LDMs but these are predominantly carried out using in vitro cell cultures or mouse models^81,125^. However, the method of excretion of LDMs from vital organs and the potential risks posed by LDMs aggregating within the body still needs to be examined futher^109,125^.

Within the published literature, most LDMs have only been tested against a few key bacterial strains. The most common models are *S. aureus* for Gram-positive and *E. coli* for Gram-negative bacteria, which are human pathogens capable of significant morbidity and have several documented drug-resistant strains^48,79^. Often in biological studies, fungal cells are overlooked, even though they pose a similar health threat^5^. This is important as fungal cells are larger than bacteria cells and possess different membrane structures and hence can be impacted differently by the antimicrobial mechanisms generated by LDMs^22^. Another limitation of the current microbial testing is the lack of “real world” scenarios. For example, if biofilm prevention is tested, typically only single strain models are used with limited testing on biofilms containing multiple bacterial strains, which are common on implant-associated infections^12^.

## Future outlook

LDMs^1,35,45,156^ utilise a combination of chemical and physical modes of action to kill pathogenic microbes with extremely high efficacy in a range of conditions. Combining this with the emerging capability to control the properties of LDMs offers an unprecedented opportunity for the research community to explore a plethora of potential antimicrobial applications. Furthermore, the synthesis of composites LDMs which can have synergistic effects provides the basis to create new paradigms in a field of antimicrobials, which has stagnated to a dangerous point^24,71^.

Importantly, there is a lot more work that needs to be done. Many facets of the antimicrobial mechanisms of LDMs remain unclear, and the library of prospective materials should be expanded. Further, the clinical and commercial applications of these materials remain under researched. Such areas of research need to be further investigated for LDMs to be considered a serious alternative to current antimicrobial treatment strategies. It is hoped that this review will provide a foundation for informed decisions and design parameters of next-generation antimicrobial LDMs with antipathogenic activity and reveal an antipathogenic technology capable of combatting AMR pathogens.

## Supplementary information

Supplementary Information

## References

[1] Interagency Coordination Group on Antimicrobial Resistance (2019). No time to wait: securing the future from drug-resistant infections.

[2] Boyce K, Morrissey O, Idnurm A, Macreadie I (2019). Insights into the global emergence of antifungal drug resistance. Microbiol. Aust.

[3] Cave R, Misra R, Chen J, Wang S, Mkrtchyan HV (2019). Whole genome sequencing revealed new molecular characteristics in multidrug resistant *staphylococci* recovered from high frequency touched surfaces in London. Sci. Rep..

[4] World Health Organization. *Prioritization of pathogens to guide discovery, research and development of new antibiotics for drug-resistant bacterial infections, including tuberculosis* (World Health Organization, 2017).

[5] Humphreys, G. & Fleck, F. in *Bulletin of the World Health Organization* Vol. 94 638+ (2016).

[6] Tan L (2018). In situ disinfection through photoinspired radical oxygen species storage and thermal-triggered release from black phosphorous with strengthened chemical stability. Small.

[7] Richter AP (2015). An environmentally benign antimicrobial nanoparticle based on a silver-infused lignin core. Nat. Nanotechnol..

[8] Jenkins J (2020). Antibacterial effects of nanopillar surfaces are mediated by cell impedance, penetration and induction of oxidative stress. Nat. Commun..

[9] Christofferson AJ (2020). Conformationally tuned antibacterial oligomers target the peptidoglycan of Gram-positive bacteria. J. Coll. Interface Sci..

[10] Rai M, Yadav A, Gade A (2009). Silver nanoparticles as a new generation of antimicrobials. Biotechnol. Adv..

[11] Durán N (2016). Silver nanoparticles: a new view on mechanistic aspects on antimicrobial activity. Nanomedicine.

[12] Kim E-J (2019). Thorn-like TiO_2_ nanoarrays with broad spectrum antimicrobial activity through physical puncture and photocatalytic action. Sci. Rep..

[13] Singh R (2019). Nanosheet and nanosphere morphology dominated photocatalytic & antibacterial properties of ZnO nanostructures. Solid State Sci.

[14] Yu X (2017). Fluorine-free preparation of titanium carbide MXene quantum dots with high near-infrared photothermal performances for cancer therapy. Nanoscale.

[15] Hebeish AA, Abdelhady MM, Youssef AM (2013). TiO_2_ nanowire and TiO_2_ nanowire doped Ag-PVP nanocomposite for antimicrobial and self-cleaning cotton textile. Carbohydr. Polym..

[16] Zhang Y (2019). Nanotoxicity of boron nitride nanosheet to bacterial membranes. Langmuir.

[17] Ahmed T (2019). Multifunctional optoelectronics via harnessing defects in layered black phosphorus. Adv. Funct. Mater..

[18] Jiang X (2020). Stable one-dimensional single crystalline black phosphorus nanowires for gas sensing. ACS Appl. Nano Mater..

[19] Ahir M (2016). Tailored-CuO-nanowire decorated with folic acid mediated coupling of the mitochondrial-ROS generation and miR425-PTEN axis in furnishing potent anti-cancer activity in human triple negative breast carcinoma cells. Biomaterials.

[20] Lu T, Wang L, Jiang Y, liu Q, Huang C (2016). Hexagonal boron nitride nanoplates as emerging biological nanovectors and their potential applications in biomedicine. J. Mater. Chem. B.

[21] Zhang L (2019). Photon-responsive antibacterial nanoplatform for synergistic photothermal-/pharmaco-therapy of skin infection. ACS Appl. Mater. Interfaces.

[22] Elbourne A (2020). Significant enhancement of antimicrobial activity in oxygen-deficient zinc oxide nanowires. ACS Appl. Bio Mater..

[23] Alimohammadi F (2018). Antimicrobial properties of 2D MnO_2_ and MoS_2_ nanomaterials vertically aligned on graphene materials and Ti_3_C_2_ MXene. Langmuir.

[24] Arabi Shamsabadi A, Sharifian Gh,M, Anasori B, Soroush M (2018). Antimicrobial mode-of-action of colloidal Ti_3_C_2_T_x_ MXene nanosheets. ACS Sustain. Chem. Eng..

[25] Sun Z (2018). New solvent-stabilized few-layer black phosphorus for antibacterial applications. Nanoscale.

[26] Tripathy A, Nanowire-based A (2018). flexible antibacterial surface reduces the viability of drug-resistant nosocomial pathogens. ACS Appl. Nano Mater..

[27] Azzam AM, Shenashen MA, Mostafa BB, Kandeel WA, El-Safty SA (2019). Antibacterial activity of magnesium oxide nano-hexagonal sheets for wastewater remediation. Environ. Progr. Sustain. Energy.

[28] Zou X, Zhang L, Wang Z, Luo Y (2016). Mechanisms of the antimicrobial activities of graphene materials. J. Am. Chem. Soc..

[29] He X (2020). Haloperoxidase mimicry by CeO_2–x_ nanorods of different aspect ratios for antibacterial performance. ACS Sustain. Chem. Eng..

[30] Hwang GB (2020). Photobactericidal activity activated by thiolated gold nanoclusters at low flux levels of white light. Nat. Commun..

[31] Mir IA, Radhakrishanan VS, Rawat K, Prasad T, Bohidar HB (2018). Bandgap tunable AgInS based quantum dots for high contrast cell imaging with enhanced photodynamic and antifungal applications. Sci. Rep..

[32] Liu J (2017). One-step hydrothermal synthesis of photoluminescent carbon nanodots with selective antibacterial activity against. Porphyromonas gingivalis. Nanoscale.

[33] Jung J-H (2016). Defect engineering route to boron nitride quantum dots and edge-hydroxylated functionalization for bio-imaging. RSC Adv..

[34] Tang X (2019). A nanohybrid composed of MoS_2_ quantum dots and MnO_2_ nanosheets with dual-emission and peroxidase mimicking properties for use in ratiometric fluorometric detection and cellular imaging of glutathione. Microchim. Acta.

[35] Aunins TR (2019). Isolating the *Escherichia coli* transcriptomic response to superoxide generation from cadmium chalcogenide quantum dots. ACS Biomater. Sci. Eng..

[36] Xu Y, Wen W, Wu J-M (2018). Titania nanowires functionalized polyester fabrics with enhanced photocatalytic and antibacterial performances. J. Hazard. Mater..

[37] Zhu Y, Liu X, Yeung KWK, Chu PK, Wu S (2017). Biofunctionalization of carbon nanotubes/chitosan hybrids on Ti implants by atom layer deposited ZnO nanostructures. Appl. Surf. Sci..

[38] De Cesare F, Di Mattia E, Zussman E, Macagnano A (2019). A study on the dependence of bacteria adhesion on the polymer nanofibre diameter. Environ. Sci. Nano.

[39] Xu Y (2019). Multifunctional CuO nanowire mesh for highly efficient solar evaporation and water purification. ACS Sustain. Chem. Eng..

[40] Ezhilarasi AA, Vijaya JJ, Kennedy LJ, Kaviyarasu K (2020). Green mediated NiO nano-rods using *Phoenix dactylifera* (dates) extract for biomedical and environmental applications. Mater. Chem. Phys..

[41] Li X (2019). Surface treatments on titanium implants via nanostructured ceria for antibacterial and anti-inflammatory capabilities. Acta Biomater.

[42] Pardo M, Shuster-Meiseles T, Levin-Zaidman S, Rudich A, Rudich Y (2014). Low cytotoxicity of inorganic nanotubes and fullerene-like nanostructures in human bronchial epithelial cells: relation to inflammatory gene induction and antioxidant response. Environ. Sci. Technol..

[43] Wang K (2016). Fabrication and thermal stability of two-dimensional carbide Ti_3_C_2_ nanosheets. Ceram. Int..

[44] Khan H (2020). Liquid metal-based synthesis of high performance monolayer SnS piezoelectric nanogenerators. Nat. Commun..

[45] Li Z (2019). Synergistic antibacterial activity of black phosphorus nanosheets modified with titanium aminobenzenesulfanato complexes. ACS Appl. Nano Mater..

[46] Zhu C (2018). Solution‐processable two‐dimensional in_2_se_3_ nanosheets as efficient photothermal agents for elimination of bacteria. Chemistry..

[47] Pandit S, Karunakaran S, Boda SK, Basu B, De M (2016). High antibacterial activity of functionalized chemically exfoliated MoS_2_. ACS Appl. Mater. Interfaces.

[48] Cheng P, Chen Y, Yan X, Wang Y, Lang WZ (2019). Highly stable and antibacterial two‐dimensional tungsten disulfide lamellar membrane for water filtration. ChemSusChem.

[49] Kaur J (2018). Biological interactions of biocompatible and water-dispersed MoS_2_ nanosheets with bacteria and human cells. Sci. Rep..

[50] Rasool K (2016). Antibacterial activity of Ti_3_C_2_T_x_ MXene. ACS Nano.

[51] Dallavalle M, Calvaresi M, Bottoni A, Melle-Franco M, Zerbetto F (2015). Graphene can wreak havoc with cell membranes. ACS Appl. Mater. Interfaces.

[52] Mejías Carpio IE, Santos CM, Wei X, Rodrigues DF (2012). Toxicity of a polymer–graphene oxide composite against bacterial planktonic cells, biofilms, and mammalian cells. Nanoscale.

[53] Mangadlao JD (2015). On the antibacterial mechanism of graphene oxide (GO) Langmuir–Blodgett films. Chem. Commun..

[54] Li J (2014). Antibacterial activity of large-area monolayer graphene film manipulated by charge transfer. Sci. Rep..

[55] Tsao N (2002). In vitro action of carboxyfullerene. J. Antimicrob. Chemother..

[56] Chen H (2013). Broad-spectrum antibacterial activity of carbon nanotubes to human gut bacteria. Small.

[57] Akasaka T, Watari F (2009). Capture of bacteria by flexible carbon nanotubes. Acta Biomater.

[58] Liu S (2009). Sharper and faster “nano darts” kill more bacteria: a study of antibacterial activity of individually dispersed pristine single-walled carbon nanotube. ACS Nano.

[59] Wang J, Wei Y, Shi X, Gao H (2013). Cellular entry of graphene nanosheets: the role of thickness, oxidation and surface adsorption. RSC Adv.

[60] Yi X, Gao H (2015). Cell interaction with graphene microsheets: near-orthogonal cutting versus parallel attachment. Nanoscale.

[61] Titov AV, Král P, Pearson R (2010). Sandwiched graphene−membrane superstructures. ACS Nano.

[62] Tu Y (2013). Destructive extraction of phospholipids from *Escherichia coli* membranes by graphene nanosheets. Nat. Nanotechnol..

[63] Wu R (2018). Membrane destruction and phospholipid extraction by using two-dimensional MoS_2_ nanosheets. Nanoscale.

[64] Jiang W (2017). Effects of charge and surface defects of multi-walled carbon nanotubes on the disruption of model cell membranes. Sci. Total Environ..

[65] Fang FC (2011). Antimicrobial actions of reactive oxygen species. mBio.

[66] Zheng K, Setyawati MI, Leong DT, Xie J (2017). Antimicrobial gold nanoclusters. ACS Nano.

[67] Yang X (2014). Antibacterial activity of two-dimensional MoS_2_ sheets. Nanoscale.

[68] Ristic BZ (2014). Photodynamic antibacterial effect of graphene quantum dots. Biomaterials.

[69] Gui R, Jin H, Wang Z, Li J (2018). Black phosphorus quantum dots: synthesis, properties, functionalized modification and applications. Chem. Soc. Rev..

[70] Ouyang J (2018). A Black phosphorus based synergistic antibacterial platform against drug resistant bacteria. J. Mater. Chem. B.

[71] Kang S, Herzberg M, Rodrigues DF, Elimelech M (2008). Antibacterial effects of carbon nanotubes: size does matter!. Langmuir.

[72] Courtney CM (2016). Photoexcited quantum dots for killing multidrug-resistant bacteria. Nat. Mater..

[73] Wang D (2018). Iron oxide nanowire-based filter for inactivation of airborne bacteria. Environ. Sci..

[74] Tian X (2019). Photogenerated charge carriers in molybdenum disulfide quantum dots with enhanced antibacterial activity. ACS Appl. Mater. Interfaces.

[75] Qiao Y (2019). Laser-activatable CuS nanodots to treat multidrug-resistant bacteria and release copper ion to accelerate healing of infected chronic nonhealing wounds. ACS Appl. Mater. Interfaces.

[76] Hu L, Zhong H, He Z (2019). The cytotoxicities in prokaryote and eukaryote varied for CdSe and CdSe/ZnS quantum dots and differed from cadmium ions. Ecotoxicol. Environ. Saf..

[77] Venkateswarlu S, Viswanath B, Reddy AS, Yoon M (2018). Fungus-derived photoluminescent carbon nanodots for ultrasensitive detection of Hg^2+^ ions and photoinduced bactericidal activity. Sens. Actuators B.

[78] Liu J (2020). Superoxide anion: critical source of high performance antibacterial activity in Co-Doped ZnO QDs. Ceram. Int..

[79] Zheng K (2016). ZnO quantum dots modified bioactive glass nanoparticles with pH-sensitive release of Zn ions, fluorescence, antibacterial and osteogenic properties. J. Mater. Chem. B.

[80] Huang L (2018). Generation of vanadium oxide quantum dots with distinct fluorescence and antibacterial activity via a room-temperature agitation strategy. ChemNanoMat.

[81] Ma W (2020). Bienzymatic synergism of vanadium oxide nanodots to efficiently eradicate drug-resistant bacteria during wound healing in vivo. J. Coll. Interface Sci..

[82] Peng D (2018). Facile and green approach to the synthesis of boron nitride quantum dots for 2,4,6-trinitrophenol sensing. ACS Appl. Mater. Interfaces.

[83] Xue Q (2017). Photoluminescent Ti_3_C_2_ MXene quantum dots for multicolor cellular imaging. Adv. Mater..

[84] Mohid SA (2019). Application of tungsten disulfide quantum dot-conjugated antimicrobial peptides in bio-imaging and antimicrobial therapy. Coll. Surf. B.

[85] Hameed ASH (2016). In vitro antibacterial activity of ZnO and Nd doped ZnO nanoparticles against ESBL producing *Escherichia coli* and *Klebsiella pneumoniae*. Sci. Rep..

[86] Karim MN (2018). Visible-light-triggered reactive-oxygen-species-mediated antibacterial activity of peroxidase-mimic CuO nanorods. ACS Appl. Nano Mater..

[87] Podder S (2018). Effect of morphology and concentration on crossover between antioxidant and pro-oxidant activity of MgO nanostructures. Inorg. Chem..

[88] Yousefi A, Ebrahimi S, Seyfoori A, Mahmoodzadeh Hosseini H (2018). Maghemite nanorods and nanospheres: synthesis and comparative physical and biological properties. BioNanoScience.

[89] Bai X, Wang L, Zong R, Zhu Y (2013). Photocatalytic activity enhanced via g-C_3_N_4_ nanoplates to nanorods. J. Phys. Chem. C.

[90] Maria Nithya JS, Pandurangan A (2014). Aqueous dispersion of polymer coated boron nitride nanotubes and their antibacterial and cytotoxicity studies. RSC Adv.

[91] Zhang X, Zhou F, Pan W, Liang Y, Wang R (2018). General construction of molybdenum-based nanowire arrays for pH-universal hydrogen evolution electrocatalysis. Adv. Funct. Mater..

[92] Ajori S, Ameri A, Ansari R (2019). The mechanical properties and structural instability of single- and double-walled boron-nitride nanotubes functionalized with 2-methoxy-N,N-dimethylethanamine (MDE) using molecular dynamics simulations. Eur. Phys. J. D.

[93] Watts MC (2019). Production of phosphorene nanoribbons. Nature.

[94] Iqbal T, Khan MA, Mahmood H (2018). Facile synthesis of ZnO nanosheets: structural, antibacterial and photocatalytic studies. Mater. Lett..

[95] Liang Y (2020). Injectable antimicrobial conductive hydrogels for wound disinfection and infectious wound healing. Biomacromolecules.

[96] Prasad K (2017). Synergic bactericidal effects of reduced graphene oxide and silver nanoparticles against Gram-positive and Gram-negative bacteria. Sci. Rep..

[97] Barbolina I (2016). Purity of graphene oxide determines its antibacterial activity. 2D Mater..

[98] Thurston JH, Hunter NM, Wayment LJ, Cornell KA (2017). Urea-derived graphitic carbon nitride (u-g-C_3_N_4_) films with highly enhanced antimicrobial and sporicidal activity. J. Coll. Interface Sci..

[99] Cui H (2019). Stimulating antibacterial activities of graphitic carbon nitride nanosheets with plasma treatment. Nanoscale.

[100] Fakhri A, Azad M, Tahami S (2017). Degradation of toxin via ultraviolet and sunlight photocatalysis using ZnO quantum dots/CuO nanosheets composites: preparation and characterization studies. J. Mater. Sci..

[101] Rajivgandhi G, Maruthupandy M, Muneeswaran T, Anand M, Manoharan N (2018). Antibiofilm activity of zinc oxide nanosheets (ZnO NSs) using *Nocardiopsis* sp. GRG1 (KT235640) against MDR strains of gram negative *Proteus mirabilis* and *Escherichia coli*. Process Biochem..

[102] Ruangtong J, T-Thienprasert J, T-Thienprasert NP (2020). Green synthesized ZnO nanosheets from banana peel extract possess anti-bacterial activity and anti-cancer activity. Mater. Today Commun..

[103] Appel JH (2016). Low cytotoxicity and genotoxicity of two-dimensional MoS_2_ and WS_2_. ACS Biomater. Sci. Eng..

[104] Gu Z, Li W, Hong L, Zhou R (2016). Exploring biological effects of MoS_2_ nanosheets on native structures of α-helical peptides. J. Chem. Phys..

[105] Navale GR (2015). Oxidative and membrane stress-mediated antibacterial activity of WS_2_ and RGO-WS_2_ nanosheets. RSC Adv..

[106] Liu X, Duan G, Li W, Zhou Z, Zhou R (2017). Membrane destruction-mediated antibacterial activity of tungsten disulfide (WS_2_). RSC Adv.

[107] Kim TI (2017). Antibacterial activities of graphene oxide–molybdenum disulfide nanocomposite films. ACS Appl. Mater. Interfaces.

[108] Kiani F (2018). Effect of graphene oxide nanosheets on visible light-assisted antibacterial activity of vertically-aligned copper oxide nanowire arrays. J. Coll. Interface Sci..

[109] Okyay TO (2015). Antibacterial properties and mechanisms of toxicity of sonochemically grown ZnO nanorods. RSC Adv..

[110] Lu X (2017). Enhanced antibacterial activity through the controlled alignment of graphene oxide nanosheets. Proc. Natl Acad. Sci. USA.

[111] Zhao C (2019). Nitrogen-doped carbon quantum dots as an antimicrobial agent against *Staphylococcus* for the treatment of infected wounds. Coll. Surf. B.

[112] Sun H, Gao N, Dong K, Ren J, Qu X (2014). Graphene quantum dots-band-aids used for wound disinfection. ACS Nano.

[113] Garcia IM, Leitune VCB, Visioli F, Samuel SMW, Collares FM (2018). Influence of zinc oxide quantum dots in the antibacterial activity and cytotoxicity of an experimental adhesive resin. J. Dent..

[114] Owusu EGA (2020). Synergistic interactions of cadmium-free quantum dots embedded in a photosensitised polymer surface: efficient killing of multidrug-resistant strains at low ambient light levels. Nanoscale.

[115] Midya L, Patra AS, Banerjee C, Panda AB, Pal S (2019). Novel nanocomposite derived from ZnO/CdS QDs embedded crosslinked chitosan: an efficient photocatalyst and effective antibacterial agent. J. Hazard. Mater..

[116] Li R (2019). Graphitic carbon nitride (g-C_3_N_4_) nanosheets functionalized composite membrane with self-cleaning and antibacterial performance. J. Hazard. Mater..

[117] Zhao H, Chen S, Quan X, Yu H, Zhao H (2016). Integration of microfiltration and visible-light-driven photocatalysis on g-C_3_N_4_ nanosheet/reduced graphene oxide membrane for enhanced water treatment. Appl. Catal. B.

[118] Rasool K (2017). Efficient antibacterial membrane based on two-dimensional Ti_3_C_2_T_x_ (MXene) nanosheets. Sci. Rep..

[119] Harame DL (1995). Si/SiGe epitaxial-base transistors. II. Process integration and analog applications. IEEE Trans. Electron Dev..

[120] Khan K (2020). Recent developments in emerging two-dimensional materials and their applications. J. Mater. Chem. C.

[121] Iannaccone G, Bonaccorso F, Colombo L, Fiori G (2018). Quantum engineering of transistors based on 2D materials heterostructures. Nat. Nanotechnol..

[122] Kargupta R (2014). Coatings and surface modifications imparting antimicrobial activity to orthopedic implants. WIREs Nanomed. Nanobiotechnol..

[123] Yadav P, Nishanthi ST, Purohit B, Shanavas A, Kailasam K (2019). Metal-free visible light photocatalytic carbon nitride quantum dots as efficient antibacterial agents: an insight study. Carbon.

[124] Heo NS (2019). Shape-controlled assemblies of graphitic carbon nitride polymer for efficient sterilization therapies of water microbial contamination via 2D g-C_3_N_4_ under visible light illumination. Mater. Sci. Eng. C.

[125] Bai X (2017). Ultrasmall WS_2_ quantum dots with visible fluorescence for protection of cells and animal models from radiation-induced damages. ACS Biomater. Sci. Eng..

[126] Mu X (2017). Black phosphorus quantum dot induced oxidative stress and toxicity in living cells and mice. ACS Appl. Mater. Interfaces.

[127] Zhang K, Feng Y, Wang F, Yang Z, Wang J (2017). Two dimensional hexagonal boron nitride (2D-hBN): synthesis, properties and applications. J. Mater. Chem. C.

[128] Ikram M (2020). Evaluation of antibacterial and catalytic potential of copper-doped chemically exfoliated boron nitride nanosheets. Ceram. Int..

[129] Xu X (2016). Synthesis of Cu_2_O octadecahedron/TiO_2_ quantum dot heterojunctions with high visible light photocatalytic activity and high stability. ACS Appl. Mater. Interfaces.

[130] Kuriakose S (2018). Black phosphorus: ambient degradation and strategies for protection. 2D Mater..

[131] Shaw ZL (2019). Broad-spectrum solvent-free layered black phosphorus as a rapid action antimicrobial. ACS Appl. Mater. Interfaces.

[132] Zhu C (2018). Solution-processable two-dimensional In_2_Se_3_ nanosheets as efficient photothermal agents for elimination of bacteria. Chemistry.

[133] Singh AK (2018). Green synthesis, characterization and antimicrobial activity of zinc oxide quantum dots using *Eclipta alba*. Mater. Chem. Phys..

[134] Vimbela GV, Ngo SM, Fraze C, Yang L, Stout DA (2017). Antibacterial properties and toxicity from metallic nanomaterials. Int. J. Nanomed.

[135] Wang W (2016). Development of novel implants with self-antibacterial performance through *in-situ* growth of 1D ZnO nanowire. Coll. Surf. B.

[136] Garcia IM, Souza VS, Hellriegel C, Scholten JD, Collares FM (2019). Ionic liquid–stabilized titania quantum dots applied in adhesive resin. J. Dent Res..

[137] Lim GP (2020). Synthesis, characterization and antifungal property of Ti_3_C_2_T_x_ MXene nanosheets. Ceram. Int..

[138] Boateng J, Catanzano O (2015). Advanced therapeutic dressings for effective wound healing—a review. J. Pharmaceut. Sci.

[139] Cheeseman S (2020). Antimicrobial metal nanomaterials: from passive to stimuli-activated applications. Adv. Sci..

[140] Biswas A, Salunke G, Khandelwal P, Das R, Poddar P (2017). Surface disordered rutile TiO_2_–graphene quantum dot hybrids: a new multifunctional material with superior photocatalytic and biofilm eradication properties. N. J. Chem.

[141] Wang R (2018). Mechanism insight into rapid photocatalytic disinfection of *Salmonella* based on vanadate QDs-interspersed g-C_3_N_4_ heterostructures. Appl. Catal. B.

[142] Sibilo R (2020). Direct and fast assessment of antimicrobial surface activity using molecular dynamics simulation and time-lapse imaging. Anal. Chem..

[143] Duan G (2015). Protein corona mitigates the cytotoxicity of graphene oxide by reducing its physical interaction with cell membrane. Nanoscale.

[144] Zhang W (2020). Cobalt-directed assembly of antibodies onto metal–phenolic networks for enhanced particle targeting. Nano Lett..

[145] Truong VK (2017). Three-dimensional organization of self-encapsulating *Gluconobacter oxydans* bacterial cells. ACS Omega.

[146] Ley, K. et al. Surface-water interface induces conformational changes critical for protein adsorption: implications for monolayer formation of EAS hydrophobin. *Front. Mol. Biosci*. **2**. 10.3389/fmolb.2015.00064 (2015). 10.3389/fmolb.2015.00064 PMC4644811 26636091

[147] Zhang Y, Xia W, Wu Y, Zhang P (2019). Prediction of MXene based 2D tunable band gap semiconductors: GW quasiparticle calculations. Nanoscale.

[148] McDougall NL, Partridge JG, Nicholls RJ, Russo SP, McCulloch DG (2017). Influence of point defects on the near edge structure of hexagonal boron nitride. Phys. Rev. B.

[149] Kistanov AA, Cai Y, Zhou K, Dmitriev SV, Zhang Y-W (2016). The role of H_2_O and O_2_ molecules and phosphorus vacancies in the structure instability of phosphorene. 2D Mater.

[150] Rajan AC (2018). Machine-learning-assisted accurate band gap predictions of functionalized MXene. Chem. Mater..

[151] Tawfik SA (2019). Efficient prediction of structural and electronic properties of hybrid 2D materials using complementary DFT and machine learning approaches. Adv. Theory Simul..

[152] Le TC (2016). An experimental and computational approach to the development of ZnO nanoparticles that are safe by design. Small.

[153] Onyszko M (2020). Few layered oxidized h-BN as nanofiller of cellulose-based paper with superior antibacterial response and enhanced mechanical/thermal performance. Int. J. Mol. Sci..

[154] Mao HY (2013). Graphene: promises, facts, opportunities, and challenges in nanomedicine. Chem. Rev..

[155] Shao J (2018). Black-phosphorus-incorporated hydrogel as a sprayable and biodegradable photothermal platform for postsurgical treatment of cancer. Adv. Sci..

[156] Zhan Y (2017). A facile and one-pot synthesis of fluorescent graphitic carbon nitride quantum dots for bio-imaging applications. N. J. Chem..

[157] Wang X, Sun G, Li N, Chen P (2016). Quantum dots derived from two-dimensional materials and their applications for catalysis and energy. Chem. Soc. Rev..

[158] Tang X (2018). Fluorination-enhanced ambient stability and electronic tolerance of black phosphorus quantum dots. Adv. Sci..

[159] Shin YC (2018). Application of black phosphorus nanodots to live cell imaging. Biomater. Res..

[160] Lin L (2014). Fabrication and luminescence of monolayered boron nitride quantum dots. Small.

[161] Liu M (2017). Boron nitride quantum dots with solvent-regulated blue/green photoluminescence and electrochemiluminescent behavior for versatile applications. Adv. Opt. Mater..

[162] Chan M-H (2016). Near-infrared light-mediated photodynamic therapy nanoplatform by the electrostatic assembly of upconversion nanoparticles with graphitic carbon nitride quantum dots. Inorg. Chem..

[163] Dong G, Zhang Y, Pan Q, Qiu J (2014). A fantastic graphitic carbon nitride (g-C_3_N_4_) material: electronic structure, photocatalytic and photoelectronic properties. J. Photochem. Photobiol. C.

[164] Wang T, Nie C, Ao Z, Wang S, An T (2020). Recent progress in g-C_3_N_4_ quantum dots: synthesis, properties and applications in photocatalytic degradation of organic pollutants. J. Mater. Chem. A.

[165] He X (2019). e*t* al. One-pot exfoliation of graphitic C_3_N_4_ quantum dots for blue QLEDs by methylamine intercalation. Small.

[166] Wang Z (2017). Understanding the aqueous stability and filtration capability of MoS_2_ membranes. Nano Lett..

[167] Martincová J, Otyepka M, Lazar P (2017). Is single layer MoS_2_ stable in the air?. Chemistry.

[168] Tan C (2018). Preparation of high-percentage 1T-phase transition metal dichalcogenide nanodots for electrochemical hydrogen evolution. Adv. Mater..

[169] Habib T (2019). Oxidation stability of Ti_3_C_2_T_x_ MXene nanosheets in solvents and composite films. npj 2D Mater. Appl..

[170] Chen X (2018). Ratiometric photoluminescence sensing based on Ti_3_C_2_ MXene quantum dots as an intracellular pH sensor. Nanoscale.

[171] Patra MK (2009). Synthesis of stable dispersion of ZnO quantum dots in aqueous medium showing visible emission from bluish green to yellow. J. Luminesc..

[172] Diamanti MV, Codeluppi S, Cordioli A, Pedeferri MP (2009). Effect of thermal oxidation on titanium oxides’ characteristics. J. Exp. Nanosci..

[173] Das R, Ali ME, Hamid SBA, Ramakrishna S, Chowdhury ZZ (2014). Carbon nanotube membranes for water purification: A bright future in water desalination. Desalination.

[174] Chen C-W, Lee M-H, Clark SJ (2004). Band gap modification of single-walled carbon nanotube and boron nitride nanotube under a transverse electric field. Nanotechnology.

[175] Bhati A, Singh A, Tripathi KM, Sonkar SK (2016). Sunlight-induced photochemical degradation of methylene blue by water-soluble carbon nanorods. Int. J. Photoenergy.

[176] Hu T, Hashmi A, Hong J (2015). Geometry, electronic structures and optical properties of phosphorus nanotubes. Nanotechnology.

[177] Hao W, Marichy C, Brioude A (2017). Promising properties of ALD boron nitride nanotube mats for water purification. Environ. Sci..

[178] Li X (2013). Boron nitride nanotubes functionalized with mesoporous silica for intracellular delivery of chemotherapy drugs. Chem. Communications..

[179] Zeng Y (2018). Scalable one-step production of porous oxygen-doped g-C3N4 nanorods with effective electron separation for excellent visible-light photocatalytic activity. Appl. Catal. B.

[180] Luo W (2019). Three-dimensional network structure assembled by g-C_3_N_4_ nanorods for improving visible-light photocatalytic performance. Appl. Catal. B.

[181] Maharaj D, Bhushan B (2013). Effect of MoS_2_ and WS_2_ nanotubes on nanofriction and wear reduction in dry and liquid environments. Tribol. Lett..

[182] Kumar R, Goel N, Kumar M (2018). High performance NO_2_ sensor using MoS_2_ nanowires network. Appl. Phys. Lett..

[183] Panchu SJ, Adebisi MA, Manikandan E, Moodley MK (2020). Catalyst-free growth of MoS_2_ nanorods synthesized by dual pulsed laser-assisted chemical vapor deposition and their structural, optical and electrical properties. J. Electron. Mater..

[184] Zhao X, Wang P, Yan Z, Ren N (2015). Room temperature photoluminescence properties of CuO nanowire arrays. Opt. Mater..

[185] Zhang W (2020). Wearable battery-free perspiration analyzing sites based on sweat flowing on ZnO nanoarrays. Nano-Micro Lett..

[186] Bai H, Liu Z, Liu L, Sun DD (2013). Large-scale production of hierarchical TiO_2_ nanorod spheres for photocatalytic elimination of contaminants and killing bacteria. Chemistry.

[187] Shyue J-J, Cochran RE, Padture NP (2006). Transparent-conducting, gas-sensing nanostructures (nanotubes, nanowires, and thin films) of titanium oxide synthesized at near-ambient conditions. J. Mater. Res..

[188] Park E-J (2014). Comparison of toxicity of different nanorod-type TiO_2_ polymorphs in vivo and in vitro. J. Appl. Toxicol..

[189] Kim HS, Oweida TJ, Yingling YG (2018). Interfacial stability of graphene-based surfaces in water and organic solvents. Journal of Materials Science.

[190] Nan HY (2013). The thermal stability of graphene in air investigated by Raman spectroscopy. J. Raman Spectrosc..

[191] Schwierz F (2010). Graphene transistors. Nat. Nanotechnol..

[192] Kim J (2015). Direct exfoliation and dispersion of two-dimensional materials in pure water via temperature control. Nat. Commun..

[193] Chang H (2013). Regulating infrared photoresponses in reduced graphene oxide phototransistors by defect and atomic structure control. ACS Nano.

[194] Walia S (2016). Defining the role of humidity in the ambient degradation of few-layer black phosphorus. 2D Mater..

[195] Czarniewska E (2019). Non-cytotoxic hydroxyl-functionalized exfoliated boron nitride nanoflakes impair the immunological function of insect haemocytes in vivo. Sci. Rep..

[196] Liu G (2018). Comparative study of pure g-C_3_N_4_ and sulfur-doped g-C_3_N_4_ catalyst performance in photo-degradation of persistent pollutant under visible light. J. Nanosci. Nanotechnol..

[197] Zhang X, Lai Z, Tan C, Zhang H (2016). Solution-processed two-dimensional MoS_2_ nanosheets: preparation, hybridization, and applications. Angew. Chem. Int. Ed.

[198] Choi W (2017). Recent development of two-dimensional transition metal dichalcogenides and their applications. Mater. Today.

[199] Vimala K, Shanthi K, Sundarraj S, Kannan S (2017). Synergistic effect of chemo-photothermal for breast cancer therapy using folic acid (FA) modified zinc oxide nanosheet. J. Coll. Interface Sci..

[200] Xu H, Yang X, Li G, Zhao C, Liao X (2015). Green synthesis of fluorescent carbon dots for selective detection of tartrazine in food samples. J. Agric. Food Chem..

[201] Seo S (2018). Black phosphorus quantum dot-based field-effect transistors with ambipolar characteristics. Appl. Surf. Sci..

[202] Lei Z, Xu S, Wan J, Wu P (2015). Facile preparation and multifunctional applications of boron nitride quantum dots. Nanoscale.

[203] Moussodia R-O, Balan L, Merlin C, Mustin C, Schneider R (2010). Biocompatible and stable ZnO quantum dots generated by functionalization with siloxane-core PAMAM dendrons. J. Mater. Chem..

[204] Solanki V (2015). Room temperature superparamagnetism in rutile TiO_2_ quantum dots produced via ECR sputtering. Nucl. Instrum. Methods Phys. Res. Sect. B.

[205] Zhang S (2017). Arrays of horizontal carbon nanotubes of controlled chirality grown using designed catalysts. Nature.

[206] Lin J (2016). Ultrafine porous boron nitride nanofibers synthesized via a freeze-drying and pyrolysis process and their adsorption properties. RSC Adv..

[207] Xu XG (2014). One-dimensional surface phonon polaritons in boron nitride nanotubes. Nat. Commun..

[208] Yang L, May PW, Yin L, Smith JA, Rosser KN (2007). Ultra fine carbon nitride nanocrystals synthesized by laser ablation in liquid solution. J. Nanopar. Res.

[209] Xu H (2016). Oscillating edge states in one-dimensional MoS_2_ nanowires. Nat. Commun..

[210] Li Z, Xiong Y, Xie Y (2003). Selected-control synthesis of ZnO nanowires and nanorods via a PEG-assisted route. Inorg. Chem..

[211] Ziąbka M (2020). Antibacterial composite hybrid coatings of veterinary medical implants. Mater. Sci. Eng. C.

[212] Wang G (2008). Facile synthesis and characterization of graphene nanosheets. J. Phys. Chem. C.

[213] Dreyer DR, Park S, Bielawski CW, Ruoff RS (2010). The chemistry of graphene oxide. Chem. Soc. Rev..

[214] Tian J (2013). Ultrathin graphitic carbon nitride nanosheets: a low-cost, green, and highly efficient electrocatalyst toward the reduction of hydrogen peroxide and its glucose biosensing application. Nanoscale.

[215] Liu G (2017). Surface modified Ti_3_C_2_ MXene nanosheets for tumor targeting photothermal/photodynamic/chemo synergistic therapy. ACS Appl. Mater. Interfaces.

[216] Wang F (2016). Nanometre-thick single-crystalline nanosheets grown at the water–air interface. Nat. Commun..

[217] Ahmed T (2017). Degradation of black phosphorus is contingent on UV–blue light exposure. npj 2D Mater. Appl..

[218] Liu N, Tang M (2020). Toxicity of different types of quantum dots to mammalian cells in vitro: an update review. J. Hazard. Mater..

[219] Venkatesan, J., Jayakumar, R., Mohandas, A., Bhatnagar, I. & Kim, S.-K. Antimicrobial activity of chitosan-carbon nanotube hydrogels. *Materials* **7**. 10.3390/ma7053946 (2014). 10.3390/ma7053946 PMC5453222 28788658

[220] Goodwin DG (2016). Biofilm development on carbon nanotube/polymer nanocomposites. Environ. Sci..

[221] Dong J, Ma Q (2015). Advances in mechanisms and signaling pathways of carbon nanotube toxicity. Nanotoxicology.

[222] Deng X (2007). Translocation and fate of multi-walled carbon nanotubes in vivo. Carbon.

[223] Guo X, Mei N (2014). Assessment of the toxic potential of graphene family nanomaterials. J. Food Drug Anal.

[224] Sarsam WS, Amiri A, Kazi SN, Badarudin A (2016). Stability and thermophysical properties of non-covalently functionalized graphene nanoplatelets nanofluids. Energy Convers. Manag.

[225] Zeng Z (2016). Graphene oxide quantum dots covalently functionalized PVDF membrane with significantly-enhanced bactericidal and antibiofouling performances. Sci. Rep..

[226] Ren C, Hu X, Zhou Q (2018). Graphene oxide quantum dots reduce oxidative stress and inhibit neurotoxicity in vitro and in vivo through catalase-like activity and metabolic regulation. Adv. Sci..

[227] Kumawat MK (2019). Preparation of graphene oxide-graphene quantum dots hybrid and its application in cancer theranostics. Mater. Sci. Eng. C.

[228] Chen J (2014). Graphene oxide exhibits broad-spectrum antimicrobial activity against bacterial phytopathogens and fungal conidia by intertwining and membrane perturbation. Nanoscale.

[229] Musico YLF, Santos CM, Dalida MLP, Rodrigues DF (2014). Surface modification of membrane filters using graphene and graphene oxide-based nanomaterials for bacterial inactivation and removal. ACS Sustain. Chem. Eng..

[230] Liu S (2011). Antibacterial activity of graphite, graphite oxide, graphene oxide, and reduced graphene oxide: membrane and oxidative stress. ACS Nano.

[231] Aksoy İ, Küçükkeçeci H, Sevgi F, Metin Ö, Hatay Patir I (2020). Photothermal antibacterial and antibiofilm activity of black phosphorus/gold nanocomposites against pathogenic bacteria. ACS Appl. Mater. Interfaces.

[232] Latiff NM, Teo WZ, Sofer Z, Fisher AC, Pumera M (2015). The cytotoxicity of layered black phosphorus. Chemistry.

[233] Huang X-W (2018). Water-based black phosphorus hybrid nanosheets as a moldable platform for wound healing applications. ACS Appl. Mater. Interfaces.

[234] Ferreira, C. J. et al. Antibacterial and remineralizing fillers in experimental orthodontic adhesives. *Materials* **12**10.3390/ma12040652 (2019). 10.3390/ma12040652 PMC6416618 30795577

[235] Kıvanç M (2018). Effects of hexagonal boron nitride nanoparticles on antimicrobial and antibiofilm activities, cell viability. Mater. Sci. Eng. C.

[236] Ma L (2020). In *vivo* toxicity evaluation of boron nitride nanosheets in *Bombyx mori* silkworm model. Chemosphere.

[237] Merlo A, Mokkapati VRSS, Pandit S, Mijakovic I (2018). Boron nitride nanomaterials: biocompatibility and bio-applications. Biomater. Sci..

[238] Xing L (2019). et al. A mechanically robust double-network hydrogel with high thermal responses via doping hydroxylated boron nitride nanosheets. J. Mater. Sci..

[239] Chung YJ, Lee BI, Ko JW, Park CB (2016). Photoactive g-C_3_N_4_ nanosheets for light-induced suppression of Alzheimer’s β-amyloid aggregation and toxicity. Adv. Healthc. Mater..

[240] Fakhroueian Z, Dehshiri AM, Katouzian F, Esmaeilzadeh P (2014). In vitro cytotoxic effects of modified zinc oxide quantum dots on breast cancer cell lines (MCF7), colon cancer cell lines (HT29) and various fungi. J. Nanopart Res..

[241] Yang Y (2014). Toxicity and biodistribution of aqueous synthesized ZnS and ZnO quantum dots in mice. Nanotoxicology.

[242] Ahamed M (2011). ZnO nanorod-induced apoptosis in human alveolar adenocarcinoma cells via p53, survivin and bax/bcl-2 pathways: role of oxidative stress. Nanomedicine.

[243] Wu J (2019). Enhanced physico-mechanical, barrier and antifungal properties of soy protein isolate film by incorporating both plant-sourced cinnamaldehyde and facile synthesized zinc oxide nanosheets. Coll. Surf..

[244] Lee J, Choi JS, Yoon M (2014). Fabrication of ZnO nanoplates for visible light-induced imaging of living cells. J. Mater. Chem. B.

[245] Liu P, Duan W, Wang Q, Li X (2010). The damage of outer membrane of *Escherichia coli* in the presence of TiO_2_ combined with UV light. Coll. Surf. B.

[246] Yang J-L (2016). The effect of carbon nanotubes and titanium dioxide incorporated in PDMS on biofilm community composition and subsequent mussel plantigrade settlement. Biofouling.

[247] Amna T (2013). TiO_2_ nanorods via one-step electrospinning technique: a novel nanomatrix for mouse myoblasts adhesion and propagation. Coll. Surf. B.

[248] Wu F (2020). In vitro and in vivo evaluation of antibacterial activity of polyhexamethylene guanidine (PHMG)-loaded TiO_2_ nanotubes. Biomed. Mater..

[249] Tong T (2013). Effects of material morphology on the phototoxicity of nano-TiO_2_ to bacteria. Environ. Sc. Technol..

[250] Zhang Q (2010). Low Ag-doped titanium dioxide nanosheet films with outstanding antimicrobial property. Environ. Sci. Technol..

[251] Song S-S (2014). Two dimensional TiO_2_ nanosheets: in vivo toxicity investigation. RSC Adv..

[252] Farshid B, Lalwani G, Sitharaman B (2015). In vitro cytocompatibility of one-dimensional and two–dimensional nanostructure-reinforced biodegradable polymeric nanocomposites. J. Biomed. Mater. Res. A.

[253] Lin H, Chen X, Li H, Yang M, Qi Y (2010). Hydrothermal synthesis and characterization of MoS_2_ nanorods. Mater. Lett..

[254] Basu P (2019). Defect-engineered MoS_2_ nanostructures for reactive oxygen species generation in the dark: antipollutant and antifungal performances. ACS Appl. Mater. Interfaces.

[255] Hao J (2017). In vivo long-term biodistribution, excretion, and toxicology of PEGylated transition-metal dichalcogenides MS_2_ (M = Mo, W, Ti) nanosheets. Adv. Sci..

[256] Kotsakidis JC (2019). Oxidation of monolayer WS2 in ambient is a photoinduced process. Nano Lett..

[257] Kapatel S, Mania C, Sumesh CK (2017). Salt assisted sonochemical exfoliation and synthesis of highly stable few-to-monolayer WS2 quantum dots with tunable optical properties. J. Mater. Sci..

[258] Wu Y-C, Liu Z-M, Chen J-T, Cai X-J, Na P (2017). Hydrothermal fabrication of hyacinth flower-like WS_2_ nanorods and their photocatalytic properties. Mater. Lett..

[259] Liu Y (2015). WS_2_ nanowires as a high-performance anode for sodium-ion batteries. Chemistry.

[260] Chowdhury C, Karmakar S, Datta A (2017). Monolayer group IV–VI monochalcogenides: low-dimensional materials for photocatalytic water splitting. J. Phys. Chem. C.

[261] Jastrzębska AM (2017). In vitro studies on cytotoxicity of delaminated Ti_3_C_2_ MXene. J. Hazard. Mater..

[262] Rozmysłowska-Wojciechowska A (2020). A simple, low-cost and green method for controlling the cytotoxicity of MXenes. Mater. Sci. Eng. C.

[263] Gao X (2019). Titanium carbide Ti3C2Tx (MXene) enhanced PAN nanofiber membrane for air purification. J. Membr. Sci..

