# Supplementary material for: Antipathogenic properties and applications of low-dimensional materials
Source: Nat Commun. 2021 Jun 23;12:3897. doi: 10.1038/s41467-021-23278-7 (PMC8222221; doi:10.1038/s41467-021-23278-7)
Supplement: Supplementary file 1 — Supplementary Information [file 41467_2021_23278_MOESM1_ESM.docx]

Supplementary Information

for

Antipathogenic Properties and Applications of Low-dimensional Materials

Z. L. Shaw,^1^ Sruthi Kuriakose,^1,2^ Samuel Cheeseman,^3^ Michael D. Dickey,^4^ Jan Genzer,^4^ Andrew J. Christofferson,^3^ Russell J. Crawford,^3^ Chris F. McConville,^3^ James Chapman,^3^ Vi Khanh Truong,^3,4^ Aaron Elbourne,^3,*^ and Sumeet Walia.^1,2,*^

^1^School of Engineering, RMIT University, Melbourne, Australia

^2^Functional Materials and Microsystems Research Group, MicroNano Research Facility, RMIT University, Melbourne, Australia

^3^School of Science, RMIT University, Melbourne VIC 3001, Australia

^4^Department of Chemical and Biomolecular Engineering, North Carolina State University, Raleigh, North Carolina 27695, USA

* Corresponding Authors including

Email: [sumeet.walia@rmit.edu.au](mailto:sumeet.walia@rmit.edu.au) and [aaron.elbourne@rmit.edu.au](mailto:aaron.elbourne@rmit.edu.au)

**Supplementary Information**

**ROS**

The redox potentials are instructive for the energy required to elicit the generation of ROS in solution and hence at the microbe interface. These reactions occur in an aqueous environment where dissolved oxygen (O_2_) acts as an electron acceptor, and water molecules behave as electron donors. Following the addition of a free electron, O_2_ can be reduced to the superoxide ion ^•^O_2_^–^ which can be oxidised to form singlet oxygen ^1^O_2_ or act as a precursor for the hydroxyl radical ^•^OH and hydrogen peroxide H_2_O_2_ which are powerful oxidising agents. Simultaneously, the charge vacancy can cause H_2_O oxidisation to form ^•^OH, which in-turn can dimerize to form H_2_O_2_.

**Supplementary Table 1. Redox potentials for the generation of reactive oxygen species (ROS).**

| **Half-reaction** | **Redox Couple** | **Redox Potential vs. RHE at Physiological pH 7.4 [V]^a^** |
| --- | --- | --- |
| O_2_ +e^-^ ^•^O_2_^-^ | O_2_/^•^O_2_^-^ | -0.18 |
| ^•^O_2_^-^ + 2H^+^ + e^-^ H_2_O_2_ | ^•^O_2_^-^/H_2_O_2_ | 0.87 |
| H_2_O_2_ + H^+^ + e^-^ H_2_O + ^•^OH | H_2_O_2_/^•^OH | 0.36 |
| H_2_O + H^+^ 2H^+^ + ^•^OH | H_2_O/^•^OH | 2.30 |
| O_2_ + 2H^+^ + 2e^-^ H_2_O_2_ | O_2_/H_2_O_2;_ | 0.26 |

a) Redox potentials were calculated using the method described in reference^1^ and the Nernst Equation.

**Heterostructures**

Owing to their size differences, 0D materials exhibit stronger quantum confinement and edge effects compared to their bulk counterparts^2^. The large surface to volume ratios and functionalization of these materials can be used as transporters to achieve targeted drug delivery. Moreover, the materials have distinct advantages over existing medicine carrier systems in terms of reducing the side effects of regular antibiotics. Drug adsorption efficiency is inversely proportional to the particle size and is directly proportional to the specific surface area of the adsorbent^3^.

***Layering Protocols, device fabrication, and exploitation***

The materials used to form heterostructures are usually chosen from the periodic table among the III-V, IV-IVI, II-VI groups. The material systems are stacked, altering their band structures to benefit the desired applications^4^. The multi stacking of the heterostructures has been explained as vertical stacking or horizontal stacking in Supplementary Fig. 1, as adopted from reference^5^.

**Supplementary Fig. 1 Vertical and horizontal stacking of heterostructures**^5^. **a**) Direct growth of 2D-materials-based vertical heterostructures by CVD with (i) h-BN by exploiting ammonia borane as precursor and (ii) graphene onto the as-grown h-BN layers. **b)** Growth by either CVD or MBE of individual 2D materials and subsequent dry transfer using pick and place techniques enabling, in principle, any combination of different 2D materials. **c)** The relative orientation of the different layers of 2D materials is key and mandatory to be controlled to design (i) vertically aligned and (ii) controlled twist heterostructures. **d)** Lateral heterostructures can be realized by (i) seeding an already grown 2D material template, (ii) growing a second 2D material by using the appropriate precursors and (iii) by a proper placement of seeds through either a pattern and etch process or a mask, which can allow the realization of different lateral heterostructures such as linear, zigzag and donut-like shape. **e)** (i) Direct growth of h-BN on graphene edges; (ii) SEM image showing a concentric h-BN/graphene heterostructure; (iii) optical image of a graphene/h-BN array of circles, with graphene circles embedded in an h-BN matrix.

The key to fabricating layered 2D materials is to have “lattice matching” to the substrate^5, 6^. When compared to the traditional heterostructures grown epitaxially, the 2D van der Waals heterostructures match their lattice structures because of the weak interlayer bonding. Hence, 2D layers with different lattice constants can be stacked together^7^. This allows the exploration of a range of materials for different applications. The growth/fabrication process^8^ for heterostructures is usually done by stacking the materials layer-by-layer via 1) transfer by traditional mechanical exfoliation of natural or synthetically grown crystals^9^. 2) direct growth process based on chemical process, predominantly using molecular beam epitaxy (MBE)^10^, metal oxide chemical vapour deposition (MOCVD)^11, 12^/chemical vapour deposition (CVD) techniques^13^/ atomic layer deposition (ALD)^14, 15^. 3) solution/ink-based deposition of the materials^16^. These techniques have been used to show significant progress in the growth/fabrication of heterostructures using TMDs and graphene family materials^17^.

The hybrid materials could give a combination of the antimicrobial mechanism effects as discussed earlier, enhancing the antimicrobial property of the hybrid material^18, 19, 20, 21, 22^. The characterisation and testing of the respective combinations of materials should shed light on the properties acquired by combining them to form hybrid materials. If a controllable and scalable method of combining LDMs can be established, it will provide a powerful tool allowing the use of these materials as next-generation antimicrobials. Before any clinical translation of any layered material as a biomedical tool, the key concerns that will need addressing are their long-term cytotoxic and environmental implications. However, it can be theorised that the useful biomedical and antimicrobial properties of hybrid LDMs could be developed and optimised by careful choice of the material to enhance their antimicrobial properties to provide solutions for specific clinical needs^23^.

**Supplementary References**

1. Wood PM. The potential diagram for oxygen at pH 7. *Biochemical Journal* **253**, 287-289 (1988).

2. Li BL*, et al.* Emerging 0D Transition-Metal Dichalcogenides for Sensors, Biomedicine, and Clean Energy. *Small* **13**, 1700527 (2017).

3. Dong J, Zhao Y, Wang K, Dong L. Comparative Investigation of Graphene Quantum Dots and Graphitic-phase C_3_N_4_ Nanosheets in Terms of Photoluminescence Properties and Biomedical Imaging. *ECS Transactions* **77**, 161-167 (2017).

4. Harame DL*, et al.* Si/SiGe epitaxial-base transistors. II. Process integration and analog applications. *IEEE Transactions on Electron Devices* **42**, 469-482 (1995).

5. Iannaccone G, Bonaccorso F, Colombo L, Fiori G. Quantum engineering of transistors based on 2D materials heterostructures. *Nature Nanotechnology* **13**, 183-191 (2018).

6. Bonaccorso F, Lombardo A, Hasan T, Sun Z, Colombo L, Ferrari AC. Production and processing of graphene and 2D crystals. *Materials Today* **15**, 564-589 (2012).

7. Wang X, Xia F. Stacked 2D materials shed light. *Nature Materials* **14**, 264-265 (2015).

8. Farrow RF, Parkin S, Dobson P, Neave J, Arrott A. *Thin film growth techniques for low-dimensional structures*. Springer Science & Business Media (2013).

9. Zhao Q, Wang T, Ryu YK, Frisenda R, Castellanos-Gomez A. An inexpensive system for the deterministic transfer of 2D materials. *Journal of Physics: Materials* **3**, 016001 (2020).

10. Harbison JP, Sands T, Tabatabaie N, Chan WK, Florez LT, Keramidas VG. MBE growth of AlGaAs/NiAl/AlGaAs heterostructures: A novel epitaxial III–V semiconductor/metal system. *Journal of Crystal Growth* **95**, 425-426 (1989).

11. Petroff PM, DenBaars SP. MBE and MOCVD growth and properties of self-assembling quantum dot arrays in III-V semiconductor structures. *Superlattices and Microstructures* **15**, 15 (1994).

12. Cun H*, et al.* Wafer-scale MOCVD growth of monolayer MoS_2_ on sapphire and SiO_2_. *Nano Research* **12**, 2646-2652 (2019).

13. Cai Z, Liu B, Zou X, Cheng H-M. Chemical Vapor Deposition Growth and Applications of Two-Dimensional Materials and Their Heterostructures. *Chemical Reviews* **118**, 6091-6133 (2018).

14. Lee SW, Liu Y, Heo J, Gordon RG. Creation and Control of Two-Dimensional Electron Gas Using Al-Based Amorphous Oxides/SrTiO_3_ Heterostructures Grown by Atomic Layer Deposition. *Nano Letters* **12**, 4775-4783 (2012).

15. Browning R, Kuperman N, Moon B, Solanki R. Atomic Layer Growth of InSe and Sb_2_Se_3_ Layered Semiconductors and Their Heterostructure. *Electronics* **6**, (2017).

16. Goebl JA, Black RW, Puthussery J, Giblin J, Kosel TH, Kuno M. Solution-Based II−VI Core/Shell Nanowire Heterostructures. *Journal of the American Chemical Society* **130**, 14822-14833 (2008).

17. Lv R*, et al.* Transition Metal Dichalcogenides and Beyond: Synthesis, Properties, and Applications of Single- and Few-Layer Nanosheets. *Accounts of Chemical Research* **48**, 56-64 (2015).

18. Chen C, Yu W, Liu T, Cao S, Tsang Y. Graphene oxide/WS_2_/Mg-doped ZnO nanocomposites for solar-light catalytic and anti-bacterial applications. *Solar Energy Materials and Solar Cells* **160**, 43-53 (2017).

19. Hou X, Shi T, Wei C, Zeng H, Hu X, Yan B. A 2D-2D heterojunction Bi_2_WO_6_/WS_2-x_ as a broad-spectrum bactericide: Sulfur vacancies mediate the interface interactions between biology and nanomaterials. *Biomaterials* **243**, 119937 (2020).

20. Xu X*, et al.* Synthesis of Cu_2_O Octadecahedron/TiO_2_ Quantum Dot Heterojunctions with High Visible Light Photocatalytic Activity and High Stability. *ACS Applied Materials & Interfaces* **8**, 91-101 (2016).

21. Mohan AN, B M, Panicker S. Facile synthesis of graphene-tin oxide nanocomposite derived from agricultural waste for enhanced antibacterial activity against *Pseudomonas aeruginosa*. *Scientific Reports* **9**, 4170 (2019).

22. Parra C*, et al.* Suppressing Bacterial Interaction with Copper Surfaces through Graphene and Hexagonal-Boron Nitride Coatings. *ACS Applied Materials & Interfaces* **7**, 6430-6437 (2015).

23. Kurapati R, Kostarelos K, Prato M, Bianco A. Biomedical Uses for 2D Materials Beyond Graphene: Current Advances and Challenges Ahead. *Advanced Materials* **28**, 6052-6074 (2016).
